# The GM2 Glycan Serves as a Functional Coreceptor for Serotype 1 Reovirus

**DOI:** 10.1371/journal.ppat.1003078

**Published:** 2012-12-06

**Authors:** Kerstin Reiss, Jennifer E. Stencel, Yan Liu, Bärbel S. Blaum, Dirk M. Reiter, Ten Feizi, Terence S. Dermody, Thilo Stehle

**Affiliations:** 1 Interfaculty Institute of Biochemistry, University of Tübingen, Tübingen, Germany; 2 Department of Pathology, Microbiology, and Immunology, Vanderbilt University School of Medicine, Nashville, Tennessee, United States of America; 3 Elizabeth B. Lamb Center for Pediatric Research, Vanderbilt University School of Medicine, Nashville, Tennessee, United States of America; 4 Glycosciences Laboratory, Department of Medicine, Imperial College London, London, United Kingdom; 5 Department of Pediatrics, Vanderbilt University School of Medicine, Nashville, Tennessee, United States of America; University of Michigan, United States of America

## Abstract

Viral attachment to target cells is the first step in infection and also serves as a determinant of tropism. Like many viruses, mammalian reoviruses bind with low affinity to cell-surface carbohydrate receptors to initiate the infectious process. Reoviruses disseminate with serotype-specific tropism in the host, which may be explained by differential glycan utilization. Although α2,3-linked sialylated oligosaccharides serve as carbohydrate receptors for type 3 reoviruses, neither a specific glycan bound by any reovirus serotype nor the function of glycan binding in type 1 reovirus infection was known. We have identified the oligosaccharide portion of ganglioside GM2 (the GM2 glycan) as a receptor for the attachment protein σ1 of reovirus strain type 1 Lang (T1L) using glycan array screening. The interaction of T1L σ1 with GM2 in solution was confirmed using NMR spectroscopy. We established that GM2 glycan engagement is required for optimal infection of mouse embryonic fibroblasts (MEFs) by T1L. Preincubation with GM2 specifically inhibited type 1 but not type 3 reovirus infection of MEFs. To provide a structural basis for these observations, we defined the mode of receptor recognition by determining the crystal structure of T1L σ1 in complex with the GM2 glycan. GM2 binds in a shallow groove in the globular head domain of T1L σ1. Both terminal sugar moieties of the GM2 glycan, *N*-acetylneuraminic acid and *N*-acetylgalactosamine, form contacts with the protein, providing an explanation for the observed specificity for GM2. Viruses with mutations in the glycan-binding domain display diminished hemagglutination capacity, a property dependent on glycan binding, and reduced capacity to infect MEFs. Our results define a novel mode of virus-glycan engagement and provide a mechanistic explanation for the serotype-dependent differences in glycan utilization by reovirus.

## Author Summary

Receptor utilization plays an important role in viral disease. Viruses must recognize a receptor or sometimes multiple receptors to infect a cell. Mammalian orthoreoviruses (reoviruses) serve as useful models for studies of viral receptor binding and pathogenesis. The reovirus experimental system allows manipulation of both the virus and the host to define mechanisms of viral attachment and disease. Like many viruses, reoviruses engage carbohydrate molecules on the cell-surface, but the oligosaccharide sequences bound and the function of glycan binding in infection were not known prior to this study. We used glycan array screening to determine that serotype 1 reoviruses bind ganglioside GM2 and found that this interaction is required for efficient infection of some types of cells. To better understand how reovirus engages GM2, we determined the structure of the reovirus attachment protein σ1 in complex with the GM2 glycan and defined residues that are required for functional receptor binding. Reoviruses are being tested in clinical trials for efficacy in the treatment of cancer. Cancer cells commonly have altered glycan profiles. Therefore, understanding how reoviruses engage cell-surface glycans might lead to improvements in oncolytic therapy.

## Introduction

Virus infections are initiated by attachment of the virus to target cells of susceptible hosts. Receptors facilitate attachment, determine host range, and govern susceptibility of particular cells to infection. While viral attachment can be a monophasic event, this process frequently involves multiple receptors, and adhesion strengthening is a common mechanism that facilitates virus entry [1]. Thus, a virus may interact with an attachment factor, commonly a carbohydrate, to adhere via low-affinity interaction to the cell-surface, where it then binds to an additional receptor with high affinity that leads to viral entry. The identities of the low-affinity attachment factors are not known for many viruses.

Mammalian orthoreoviruses (reoviruses) serve as highly tractable models to study virus-receptor interactions. These viruses replicate to high titer, facilitating biochemical and biophysical studies, and both the virus and host can be manipulated genetically. Reoviruses contain ten segments of double-stranded RNA (dsRNA) encapsidated within two protein shells. Reoviruses can infect the gastrointestinal and respiratory tracts of a variety of mammals but rarely cause systemic disease outside of the immediate newborn period [2]. Most children are seropositive for reovirus by the age of 5 years [3]. Reoviruses preferentially infect tumor cells and are being tested in clinical trials for the treatment of a variety of cancers [4]–[6]. It is not yet clear why reoviruses infect tumor cells more efficiently than untransformed cells, but it is likely that distribution, accessibility, and density of cellular receptors contribute to this process.

The three known reovirus serotypes are represented by the prototype strains type 1 Lang (T1L), type 2 Jones (T2J), and type 3 Dearing (T3D). These three strains differ markedly in cell tropism and viral spread, and these properties have been studied extensively using newborn mice [7]. T1L spreads hematogenously and infects ependymal cells, leading to non-lethal hydrocephalus [8], [9]. In contrast, T3D disseminates hematogenously and neurally and infects neurons, causing lethal encephalitis [7]–[12]. These serotype-dependent differences are linked to sequence variations in the σ1 outer-capsid protein [7], [9].

The σ1 protein mediates the attachment of the virus to target cells [9], [13]. It is a 150 kDa homotrimeric protein that assembles into a long fiber that protrudes from the virion surface [14]. The σ1 protein can be partitioned into three functionally and structurally distinct domains: the tail, body, and head. The N-terminal tail spans about 170 residues and is predicted to form an α-helical coiled coil [15]–[17]. The body domain comprises approximately 100 residues and primarily consists of β-spiral repeats [18], [19]. The C-terminal 150 residues fold into the compact head domain composed of eight antiparallel β-strands that assemble into a jelly-roll [18]. The head binds with high affinity to junctional adhesion molecule-A (JAM-A) [20], which serves as a receptor for all known reovirus serotypes [21]. JAM-A is a homodimeric member of the immunoglobulin superfamily [22] located in tight junctions [23].

The structure and receptor-binding properties of reovirus T3D σ1 have been studied most extensively [18], [19], [24], [25]. Interactions of T3D σ1 and JAM-A exclusively involve the σ1 head, which binds the N-terminal D1 domain of JAM-A [25], [26]. JAM-A binds with higher affinity to σ1 than to itself; thus, the engagement of σ1 to JAM-A disrupts the JAM-A homodimer. The JAM-A-binding site is highly conserved among the three reovirus serotypes; thus, it is predicted that the T1L, T2J, and T3D reovirus σ1 proteins engage JAM-A in a similar manner and with similar affinities. Although binding to JAM-A is required for hematogenous dissemination, differences in target cell selection within the CNS displayed by T1L and T3D are retained in JAM-A deficient mice inoculated with the viruses intracranially [11]. Therefore, interactions with JAM-A are unlikely to dictate the serotype-specific differences in cell tropism in the nervous system. Instead, these differences in tropism are likely a consequence of virus binding to serotype-specific receptors.

In addition to JAM-A, reoviruses bind to cell-surface glycans. However, the limited knowledge of glycan coreceptors for reovirus is an obstacle to a precise understanding of the contribution of individual receptors to viral tropism and disease. While there is considerable information about carbohydrate-mediated interactions of T3D with host cells, the role of glycan binding in other reovirus serotypes is not known. T3D σ1 interacts with α-linked 5-*N*-acetyl neuraminic acid (Neu5Ac) [19], [27], and crystal structures of T3D σ1 in complex with sialyllactose-based compounds terminating in α2,3-, α2,6-, and α2,8-linked Neu5Ac have identified the glycan-binding site [19]. The N-terminal portion of the T3D σ1 body, which lies close to the mid-point of the molecule, engages Neu5Ac via a complex network of interactions that are identical for the three linkages tested. Contacts include a bidentate salt bridge, which connects arginine 202 with the Neu5Ac carboxylate, and a number of augmenting hydrogen bonds and non-polar interactions. The additional sugar rings of the lactose backbone make minimal contacts with T3D σ1, suggesting that T3D σ1 recognizes a different carbohydrate sequence on the cell-surface [19].

Much less is known about the interaction of type 1 reovirus with cell-surface glycans. Hemagglutination is dependent on glycan-engagement, and serotypes 1 and 3 display differences in hemagglutination profiles, suggesting that they differentially engage cell-surface glycans [28]. Type 1 reoviruses agglutinate human and not bovine red blood cells, whereas type 3 reoviruses agglutinate bovine erythrocytes well and human erythrocytes less efficiently than type 1 strains [29]. Hemagglutination studies using chimeric and truncated σ1 proteins expressed in insect cells using baculovirus vectors suggest that the carbohydrate-binding site of T1L σ1 resides just beneath the head domain [27]. Additionally, neuraminidase treatment diminishes infection of intestinal M cells by T1L, suggesting that type 1 reoviruses can engage sialic acid at least in some contexts [30]. T1L reovirus does not bind to sialylated glycophorin, whereas T3D reovirus does [27], [31]. Therefore, the glycan recognized by type 1 reoviruses differs from that recognized by type 3 strains.

In this study, we employed glycan microarray analyses to identify ganglioside GM2 as a glycan receptor for reovirus T1L, and we used structural and infectivity data to define the glycan-protein interaction and the biological relevance of glycan binding to infection of host cells. Taken together, our structure-function data provide insight into how the GM2 glycan is specifically recognized by type 1 reovirus and explain the serotype-specific nature of reovirus glycan utilization.

## Results

### Infectivity of T1L reovirus is dependent on Neu5Ac

**Figure 1:**
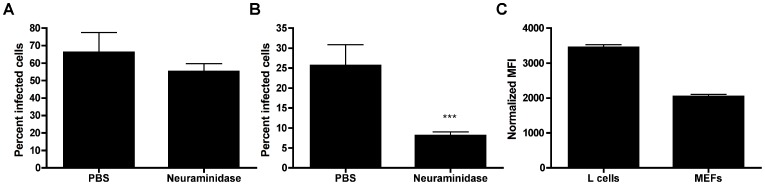
The effect of neuraminidase treatment on T1L infectivity in L cells and MEFs. (**A**) L cells or (**B**) MEFs were treated with *A. ureafaciens* neuraminidase for 1 h, followed by adsorption of T1L at an MOI of 10 or 100 PFU/cell, respectively. Cells were washed twice with PBS, and fresh medium was added. After incubation at 37°C for 20 h, cells were fixed, and reovirus antigen was detected by indirect immunofluorescence. Nuclei were stained with DAPI. The percentage of infected cells in three fields of view per well was determined. The results are expressed as the mean percent infected cells per well in triplicate wells for two independent experiments. Error bars represent standard deviations. (**A**) n.s., (**B**) ***, *P*<0.0001, as determined by two-tailed Student's *t* test. (**C**) L cells or MEFs were stained with anti-JAM-A antibody followed by Alexa-488 labeled secondary antibody to measure cell-surface JAM-A expression. Fluorescence was detected by flow cytometry. Cells were gated on forward and side scatter and the mean fluorescence intensity (MFI) of Alexa-488 was quantified. Results shown are from a representative of three experiments each done with duplicate samples.

To investigate glycan engagement by T1L, we established a cell-culture system in which glycan binding could be evaluated. Binding to sialic acid is dispensable for infection of murine L929 (L) fibroblast cells by either type 1 or type 3 reovirus [27], [32], [33]. However, sialic acid engagement is required for optimal infection of MEFs [11], [33] and HeLa cells by type 3 reoviruses [25], [27], [33]. To determine whether sialylated glycan engagement is required for efficient infection by T1L, we pretreated L cells (Figure 1A) and MEFs (Figure 1B) with *Arthrobacter ureafaciens* neuraminidase to remove cell-surface sialic acid. Neuraminidase treatment did not impair the capacity of T1L to infect L cells, as previously shown [32]. In contrast, neuraminidase treatment reduced T1L infectivity of MEFs (Figure 1B) and also HeLa cells (data not shown), suggesting that sialic acid engagement by T1L is required for optimal infection of some cell types. Of note, GM2 is expressed on MEFs [34], which display glycan-dependent infection, and L cells [35], which do not require glycan-binding for infection. While both L cells and MEFs are of murine origin, differences in sialic acid requirements are likely accounted for by differences in the expression on these cells of the known proteinaceous reovirus receptor, JAM-A. L cells, which do not require sialic acid for efficient entry, express higher levels of cell-surface JAM-A than do MEFs (Figure 1C). Thus, T1L may infect MEFs using an adhesion-strengthening mechanism in which binding to glycans must precede engagement of the relatively low abundance JAM-A receptor.

### Glycan array screening identifies GM2 as a preferred ligand for T1L σ1

To assess the carbohydrate-binding specificity of T1L reovirus, we expressed and purified recombinant hexahistidine-tagged T1L σ1 protein for binding analyses in neoglycolipid-based glycan microarrays. Based on sequence alignment with T3D σ1, for which several crystal structures exist [18], [24], [25], two constructs were designed. The first construct, σ1_long_, comprised amino acids 261–470, which were predicted to fold into three β-spiral repeats and the C-terminal head domain. The second construct, σ1_short_, comprised amino acids 300–470, which were predicted to form only the most C-terminal β-spiral and the head domain. Both σ1 constructs included the predicted carbohydrate-binding site, which was reported to lie in close proximity to the head domain [27].

Glycan microarray analyses were carried out initially with σ1_long_ using an array composed of 124 lipid-linked oligosaccharide probes. Among these are 119 sialylated probes with differing sialic acid linkages, backbone sequences, chain lengths, and branching patterns; five non-sialylated probes were included as negative controls (Table S1). The results from the glycan array screening showed a signal for the ganglioside GM2 that, despite its low intensity, was significantly stronger than the other signals (Figure S1). The GM2 glycan sequence contains two terminal sugars, Neu5Ac and *N*-acetylgalactosamine (GalNAc), that are both linked to a central galactose (Gal) via α2,3 and β1,4 linkages, respectively. The Gal is connected, via a β1,4 linkage, to a glucose (Glc), which is attached to a ceramide anchor.

**Figure 2:**
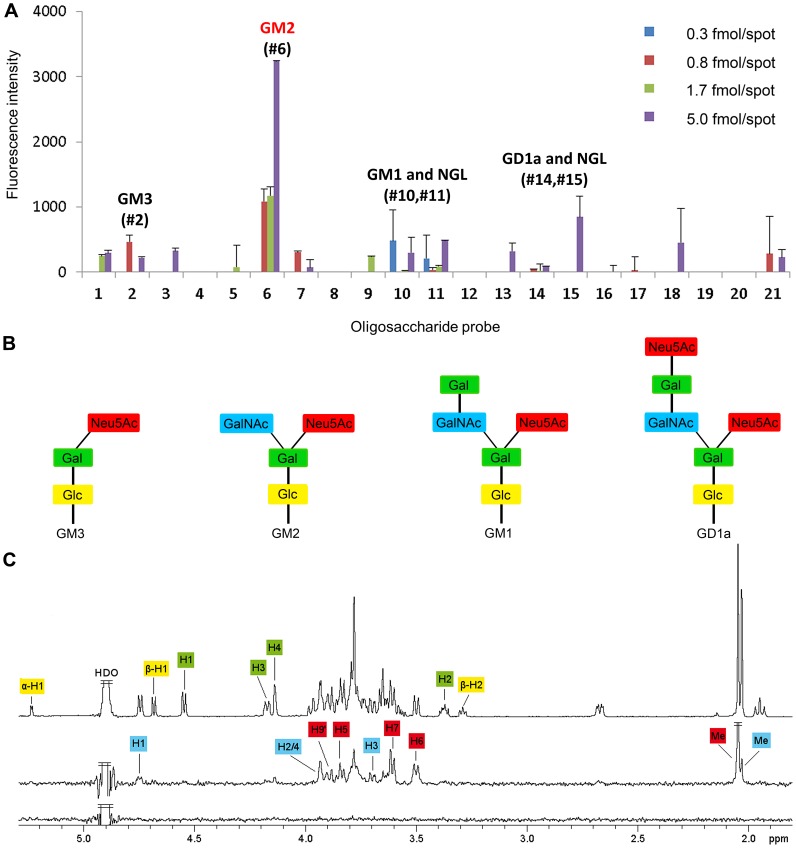
T1L reovirus uses GM2 as a coreceptor. (**A**) Glycan microarray analysis of recombinant T1L σ1_short_ using 21 lipid-linked oligosaccharide probes. Each oligosaccharide probe was arrayed at four levels (as indicated) in duplicate. Numerical scores of the binding signals are means of duplicate spots (with error bars). The complete list of probes and their sequences are provided in Table S2. (**B**) Diagrams of “a series” gangliosides GM3, GM2, GM1, and GD1a present in the glycan array. Ceramide, glucose (Glc), galactose (Gal), *N*-5-acetyl neuraminic acid (Neu5Ac), and *N*-acetylgalactosamine (GalNAc) moieties are indicated. (**C**) STD NMR spectroscopy demonstrates that T1L σ1 binds to the GM2 glycan in solution. Upper spectrum: ^1^H spectrum of the GM2 oligosaccharide alone; middle: STD spectrum of T1L σ1 and the GM2 glycan; and lower spectrum: STD spectrum of the GM2 glycan alone as a control for direct excitation of the ligand. The protons are labeled and color-coded according to the sugar moieties within the GM2 oligosaccharide. The large peak just below 3.8 ppm unites the Neu5Ac H4 and H8 and the GalNAc H6 resonances.

Additional analyses were carried out with the σ1_short_ construct, which was predicted to have less steric hindrance imposed by the long body domain and, therefore, to perhaps yield clearer results. Since the initial screen with σ1_long_ revealed GM2 as a likely carbohydrate receptor, the second array was comprised of 21 ganglioside-related saccharide probes that included GM2 (Table S2). The results from this screen confirmed binding of the protein to GM2 and yielded a higher signal-to-noise ratio than the initial screen (Figure 2A). GM2 clearly exhibited the highest signal among the probes investigated, whereas several other structurally closely related probes (Figure 2B), e.g., the “a series” gangliosides GM3, GM1, and GD1a (sequences in Table S2), elicited marginally detectable low signals. The overall binding intensity of the σ1 protein, even with the short construct, is lower than that of other proteins tested in the same arrays, e.g., the VP1 proteins of polyoma viruses JCV and SV40, and the fiber knobs of adenovirus Ad37 (data not shown).

### T1L σ1 interacts with the GM2 glycan in solution

To verify that T1L σ1 binds specifically to the GM2 glycan, we performed STD NMR spectroscopy experiments with σ1 and the glycan. This method is especially well suited to detect low-affinity binding between a large molecule, such as σ1, and a small oligosaccharide [36]–[38]. In an STD NMR experiment, the protein is selectively excited, and magnetization transfer to the ligand is observed if complex formation and rapid release of the ligand take place. If these conditions are fulfilled, the STD spectrum contains ligand resonances belonging to the binding epitope. A control experiment without protein serves to exclude direct excitation of the ligand. Using STD NMR, we found that T1L σ1 binds to the GM2 oligosaccharide in solution. Moreover, the STD analysis identified the protons of the carbohydrate that lie in close proximity (about 5 Å) to σ1 in the complex (Figure 2C, Figure S2A). All of the GM2 protons in the σ1-GM2 complex are part of the terminal Neu5Ac or the GalNAc moieties. The most prominent peak in the STD NMR spectrum belongs to the Neu5Ac methyl group, which receives considerably more saturation than the GalNAc methyl group. Protons H5, H6, H7, and one of the two H9 protons of Neu5Ac also are readily identified in the STD NMR spectrum, while the axial and equatorial H3 protons of this moiety receive little, if any, magnetization from the protein. Saturation transfer to the Neu5Ac protons H4 and H8 cannot be evaluated unambiguously because the resonances of both overlap with each other and with the GalNAc H6 resonance. Protons H1 through H4 of the GalNAc ring also are seen in the difference spectrum, although they are generally less prominent than the Neu5Ac protons. No noteworthy transfer was observed for the GM2 galactose and glucose rings. Thus, the STD NMR spectroscopy data show that the T1L σ1-GM2 glycan interaction is based on contacts with ring atoms and the glycerol side chain of Neu5Ac, with additional contacts contributed by GalNAc ring atoms. The STD NMR experiment was repeated with the linear GM3 glycan (Figure S2B), which lacks the terminal GalNAc present on GM2. The difference spectrum demonstrates that the GM3 trisaccharide interacts with T1L σ1 and that saturation transfer is observed to Neu5Ac protons only. The STD NMR experiments allow no direct estimate of relative affinities for GM2 and GM3, but it is likely that T1L σ1 binds with greater affinity to the GM2 glycan because of the additional contacts with the terminal GalNAc of this compound. This assumption is consistent with our observation that the GM2 binding signal on the microglycan array is much higher compared with the GM3 signal (Figure 2A).

### Infection of MEF cells with T1L reovirus is blocked by preincubation with the GM2 glycan

**Figure 3:**
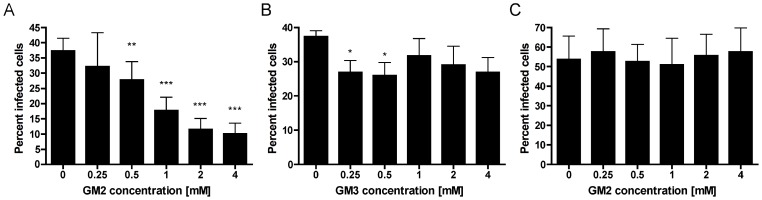
The effect of soluble glycans on T1L infectivity of MEFs. (**A,B**) T1L or (**C**) T3D (10^7^ PFU/well) were pre-incubated with the GM2 (**A,C**) or GM3 (**B**) glycan at the concentrations shown for 1 h prior to adsorption to MEFs at a final MOI of 100 PFU/cell. Cells were washed twice with PBS, and fresh medium was added. After incubation at 37°C for 20 h, cells were fixed and reovirus antigen was detected by indirect immunofluorescence. Nuclei were quantified by DAPI staining. The results are expressed as the mean percent infected cells per field in triplicate wells for two independent experiments. Error bars represent standard deviations. *, *P*<0.05; **, *P*<0.01; ***, *P*<0.0001, as determined by two-tailed Student's *t* test.

To investigate whether GM2 serves as a functional receptor for T1L reovirus, we tested the soluble GM2 glycan for the capacity to inhibit T1L infection of MEFs. Preincubation of the GM2 glycan with T1L resulted in a dose-dependent decrease in T1L infectivity (Figure 3A). However, preincubation of T1L with the GM3 glycan diminished infectivity to a lesser extent and was not dose-dependent (Figure 3B). As a specificity control, incubation of reovirus T3D with the GM2 glycan did not diminish the capacity of T3D to infect MEFs (Figure 3C). These findings demonstrate that the GM2 glycan is specifically recognized by T1L and serves as a physiologically relevant coreceptor.

### Crystal structure of T1L σ1 in complex with the GM2 glycan

**Figure 4:**
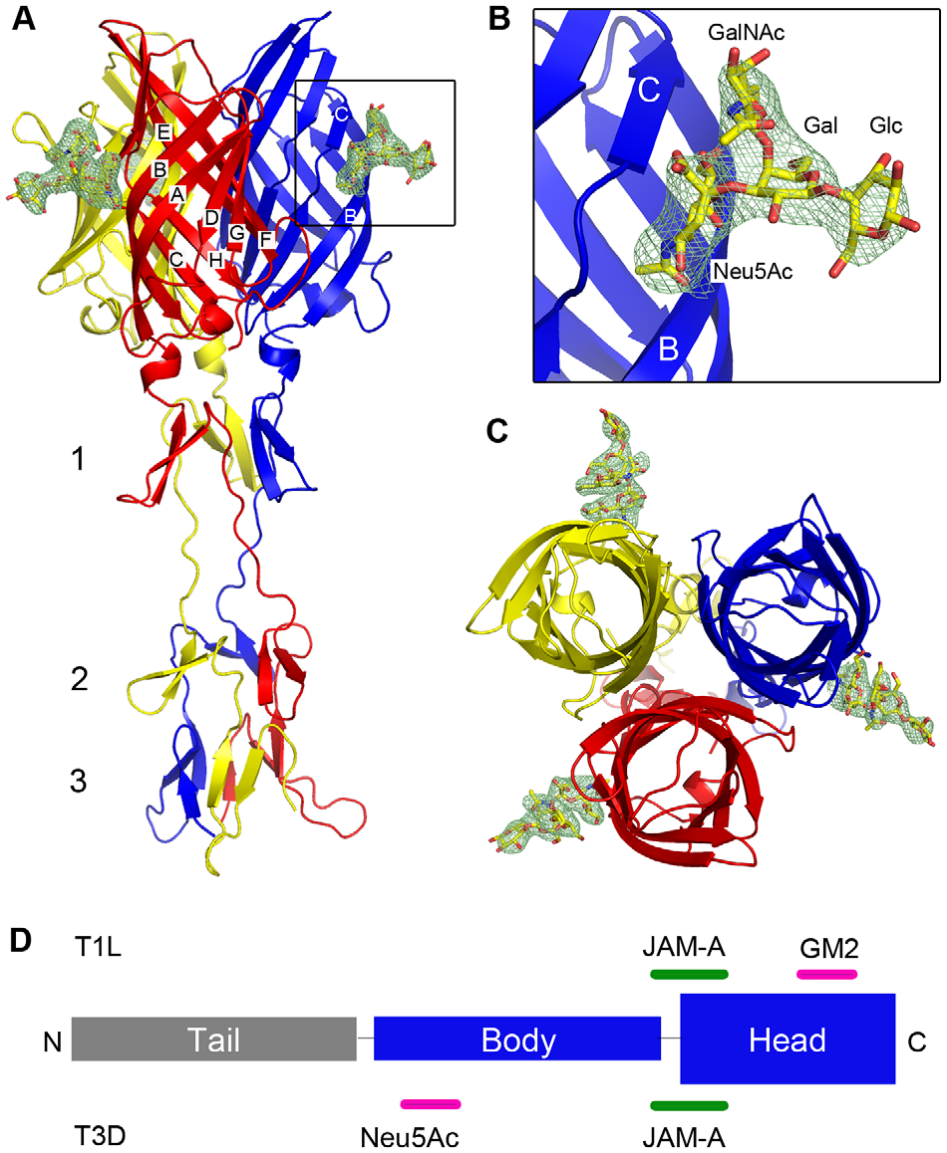
Crystal structure of T1L σ1 in complex with the GM2 glycan. Ribbon tracing of the complex viewed from the side (**A**) with a close-up of the carbohydrate-binding site (**B**) and top-view of the complex (**C**). The three T1L σ1 monomers are depicted in blue, red, and yellow. β-spiral repeats 1, 2, and 3 and β-strands A-H are labeled. The GM2 oligosaccharide is shown in stick representation, with carbons, oxygens, and nitrogens colored yellow, red, and blue, respectively. An unbiased F_o_-F_c_ map of the carbohydrate is shown at a contour level of 3 σ for 2.0 Å around the GM2 glycan (see Materials and Methods section). (**D**) Schematic representation of the σ1 domain organization. Binding sites in T1L and T3D σ1 for JAM-A and carbohydrate are depicted in green and pink, respectively.

To visualize interactions between T1L σ1 and its coreceptor, we determined the crystal structure of the σ1_long_ construct in complex with the GM2 glycan. The overall structure of the monomer and the organization of the trimer are similar to the T3D σ1 structure [18]. The crystallized T1L σ1 protein folds into three β-spiral repeats and a globular C-terminal head domain (Figure 4A–C). The head domain, comprising amino acids 327–470, is constructed from two Greek-key motifs, each consisting of four β-strands (β-strands A–D and E-H). β-spiral repeats 1 (amino acids 310–326) and 3 (residues 268 to 287) form proline-type β-turns, with both prolines being in the cis-configuration, again similar to T3D σ1. β-spiral repeat 2 (amino acids 288–305) is initiated by a serine residue (S291). In T3D σ1, threonine 278 occupies an analogous position. Both residues are non-standard, as normally only glycines or prolines are tolerated at this position [18], [39].

Although the structure has only intermediate resolution, it has good refinement statistics (Table 1). The unbiased electron density map shown in Figure 4 was determined prior to inclusion of the glycan in the refinement and therefore does not contain any information about GM2. The map has interpretable electron density for all four sugar moieties of GM2, including the unique features of Neu5Ac, in all three T1L σ1 monomers. The three copies of the glycan are crystallographically independent but nevertheless make nearly identical contacts with their respective binding pockets, providing additional support for the validity of the observed interactions. The GM2 glycan binds to the upper region of the T1L σ1 head and thus not near the β-spiral region as predicted earlier [18]. A schematic representation of the σ1 domain organization is shown in Figure 4D, including the localization of the respective binding sites for carbohydrate and JAM-A in T1L and T3D σ1.

**Table 1:** Data collection and refinement statistics for the T1L σ1-GM2 complex.

**Data collection**	
Resolution (Å)	50-3.60 (3.69-3.60)
Space group	P3_2_21
a, c (Å)	147.5, 164.5
α, β, γ (°)	90, 90, 120
R_meas_ (%)	11.5 (61.9)
CC_1/2_ (%)*	99.8 (88.1)
λ(Å)	1.0
I/σ(I)	17.2 (3.1)
Completeness (%)	99.9 (99.8)
Total reflections	151484 (11137)
Unique reflections	24422 (1782)
Redundancy	6.2
**Refinement**	
R_work_/R_free_ (%)**	18.5/20.4
B-factors	
Chain A (Å^2^)	86.6
Chain B (Å^2^)	86.9
Chain C (Å^2^)	98.7
GM2-A (complete) (Å^2^)	99.4
Neu5Ac/GalNAc-A (Å^2^)	85.9/89.0
GM2-B (complete) (Å^2^)	101.2
Neu5Ac/GalNAc-B (Å^2^)	87.9/94.3
GM2-C (complete) (Å^2^)	111.4
Neu5Ac/GalNAc-C (Å^2^)	95.2/108.3
Number of atoms	
Protein	4776
GM2 glycan	171
r.m.s.d.	
Bond lengths (Å)	0.01
Bond angles (°)	1.11
Ramachandran Plot	
Favored (%)	593 (97.5)
Allowed (%)	15 (2.5)
Outliers (%)	0

r.m.s.d. = root-mean-square deviation.

* CC_1/2_ = correlation coefficient ([90]).

** R_free_ was calculated with 10% of the data.

The Neu5Ac residue contributes the majority of the contacts between GM2 and T1L σ1 and is wedged into a shallow groove bordered on each side by β-strands B and C. Additional contacts involve the GalNAc moiety. The lactose component, which forms the backbone of the branched glycan and would be linked to the ceramide anchor in the GM2 ganglioside, points away from the protein. The mobilities of the sugar moieties are reflected in their thermal factors (B-factors). The average B-factors of Neu5Ac and GalNAc are in the same range as those of the neighboring protein residues, indicating nearly complete occupancy of the glycan-binding pockets (Table 1). The remaining two sugars, and especially the glucose moiety, have elevated B-factors, in agreement with their lack of contacts to protein residues and resultant higher mobility (Table 1).

**Figure 5:**
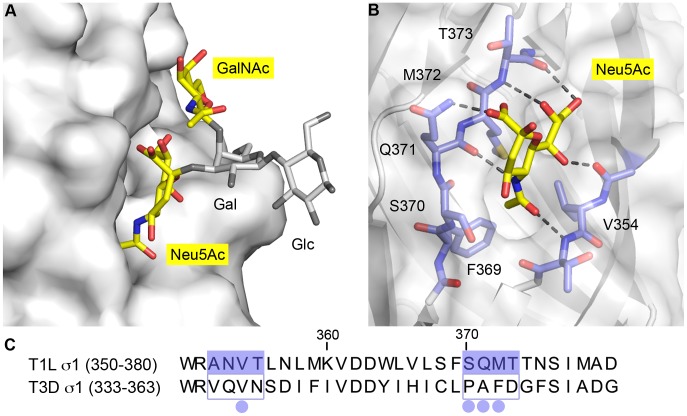
The carbohydrate-binding site of T1L σ1. (**A**) Surface representation of T1L σ1 shown in light gray. The GM2 glycan is depicted in stick representation with the two terminal sugars, Neu5Ac and GalNAc, that contact T1L σ1 shown in color, and the Gal and Glc residues shown in gray. (**B**) Close-up view of the Neu5Ac-binding pocket, with contacting residues shown in stick representation in blue (carbons) and the protein surface shown in light gray. Neu5Ac is depicted in stick representation and colored as in Figure 4. Hydrogen bonds between T1L σ1 and Neu5Ac are represented with black dashes. The methyl group of the *N*-acetyl chain of Neu5Ac inserts into a hydrophobic pocket formed by residues V354, F369, and M372. (**C**) Sequence alignment of the carbohydrate-binding site of T1L σ1 (amino acids 350–380) with the corresponding region of T3D σ1 (residues 333–363). The two β-strands forming the carbohydrate-binding site of T1L σ1 are highlighted in blue. The four residues included in the mutational analyses are marked with blue dots.

The Neu5Ac residue can be unambiguously placed in the electron density map due to unique identifying features of this sugar compound (Figure 5A, B). The *N*-acetyl and glycerol chains of Neu5Ac insert between β-strands B and C, where they form hydrogen bonds with backbone atoms of both β-strands (Figure 5A, B). Additionally, the methyl group of the Neu5Ac *N*-acetyl chain inserts into a hydrophobic pocket flanked by V354, F369, and M372, consistent with the dominance of this group in the STD NMR spectrum. The side chain of Q371 likely forms a hydrogen bond with the Neu5Ac carboxylate. However, at 3.6 Å resolution, the conformations of protein side chains cannot be unambiguously determined.

There are two possible orientations for the GalNAc group as a result of the electron density. For our crystallographic model, we selected the sugar conformation that is favored according to the corresponding *Ca*rbohydrate *R*amachandran *p*lot (CaRp) (Figure S3, Table S3) [40]. This orientation of GalNAc also is preferred by GM2 in solution as assessed by NMR spectroscopy [41]. The GalNAc moiety does not form any hydrogen bonds with T1L σ1, but it clearly interacts with the protein through van der Waals contacts (Figure 5A). Similar contacts are made for each of the two possible orientations of the GalNAc ring.

### Crystal structure of T1L σ1 in complex with the GM3 glycan

**Figure 6:**
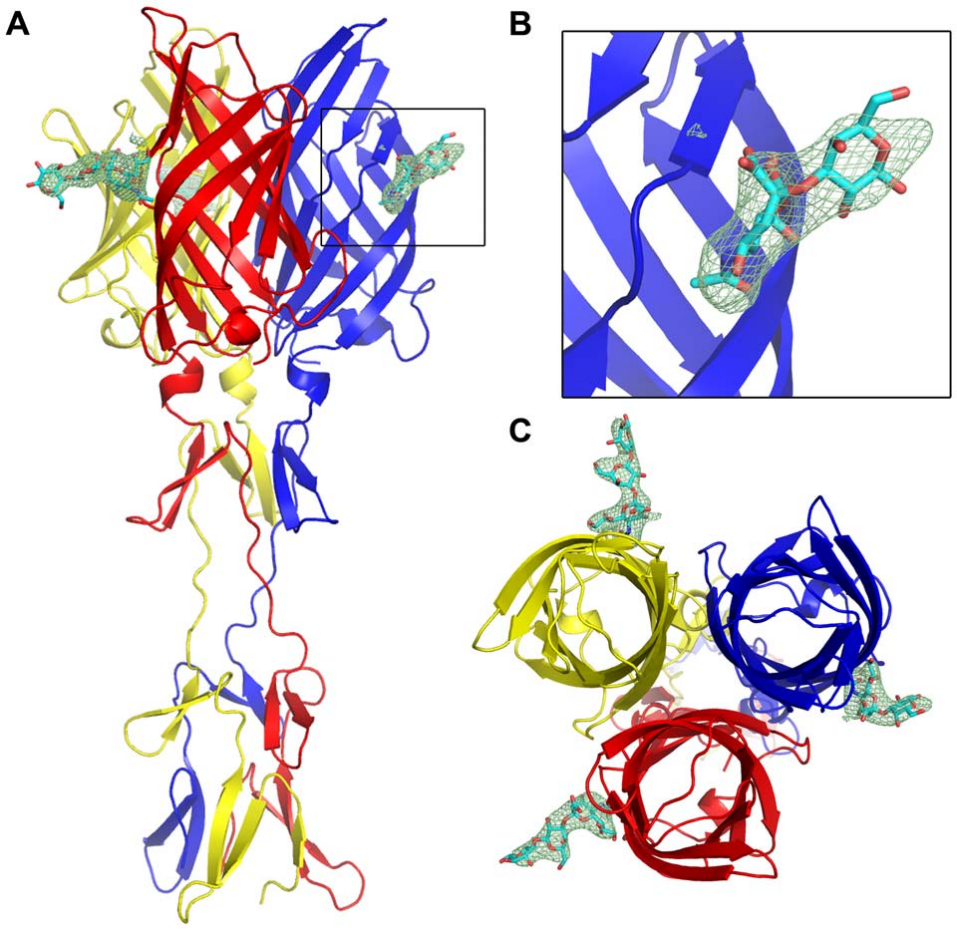
Crystal structure of T1L σ1 in complex with the GM3 glycan. Ribbon tracing of the complex viewed from the side (**A**) with a close-up of the carbohydrate-binding site (**B**) and top-view of the complex (**C**). The three σ1 monomers are depicted in blue, red, and yellow. The ligand is depicted in stick representation in cyan. An unbiased F_o_-F_c_ electron density map is shown at 3.0 σ contour level for 3 Å around the GM3 glycan.

The GM3 glycan differs from the GM2 oligosaccharide in lacking the GalNAc moiety (Figure 2B). Although GM3 exhibited only very weak binding to T1L σ1 in the glycan arrays (Figure 2A), the structure of T1L σ1 in complex with the GM2 glycan indicated that GM3 contains most of the essential features for complex formation and could potentially engage T1L σ1, albeit with lower affinity compared to GM2. We therefore determined a crystal structure of T1L σ1 in complex with the GM3 glycan at 3.5 Å resolution (Table 2). The structure shows that T1L σ1 binds to the GM3 glycan at the same site as the GM2 glycan, using identical contacts for the Neu5Ac group (Figure 6). The Neu5Ac residues of the T1L σ1-GM3 and T1L σ1-GM2 complex structures superimpose with an r.m.s.d. value of 0.76 Å (Figure S4). As is the case for the T1L σ1-GM2 complex, the lactose moiety of the GM3 glycan points away from the protein.

**Table 2:** Data collection and refinement statistics for the T1L σ1-GM3 complex.

**Data collection**	
Resolution (Å)	50-3.50 (3.59-3.50)
Space group	P3_2_21
a, c (Å)	149.4, 165.2
α, β, γ (°)	90, 90, 120
R_meas_ (%)	12.7 (64.3)
CC_1/2_ (%)*	99.6 (86.8)
λ(Å)	1.0
I/σ(I)	11.77 (3.34)
Completeness (%)	97.7 (98.7)
Total reflections	107527 (8217)
Unique reflections	26751 (1984)
Redundancy	4.0
**Refinement**	
R_work_/R_free_ (%)**	18.6/19.7
B-factors	
Chain A (Å^2^)	81.4
Chain B (Å^2^)	83.4
Chain C (Å^2^)	90.5
GM3-A (complete) (Å^2^)	104.1
Neu5Ac-A (Å^2^)	91.4
GM3-B (complete) (Å^2^)	101.4
Neu5Ac-B (Å^2^)	81.3
GM3-C (complete) (Å^2^)	103.0
Neu5Ac-C (Å^2^)	91.4
Number of atoms	
Protein	4794
GM3	118
r.m.s.d.	
Bond lengths (Å)	1.09
Bond angles (°)	0.01
Ramachandran plot	
Favored (%)	602 (98.9)
Allowed (%)	6 (1.0)
Outliers (%)	1 (0.2)

r.m.s.d. = root-mean-square deviation.

* CC_1/2_ = correlation coefficient ([90]).

** R_free_ was calculated with 10% of the data.

### Residues in T1L reovirus required for carbohydrate engagement

To identify residues in T1L σ1 required for glycan binding, we generated T1L reoviruses carrying point mutations in the GM2-binding site using plasmid-based reverse genetics [42]. Residues V354, S370, Q371, and M372 were chosen for mutational analysis, as inspection of the T1L σ1-GM2 complex structure showed that each of these residues is in close proximity to the bound glycan (Figure 5B). For point mutants V354F, V354L, and M372L, the amino acids present in T1L σ1 were replaced with residues predicted to partially block the putative Neu5Ac-binding pocket. Residue Q371 was replaced with an acidic residue to introduce a negative charge that was expected to repel the Neu5Ac moiety and interfere with binding to the GM2 glycan (Figure 5B). Point mutants S370P, Q371A, and M372F were generated to replace a T1L σ1 residue with the corresponding residue in T3D σ1, which does not bind a carbohydrate receptor via its head domain [19] (Figure 5C). The S1 genes of all mutant viruses were sequenced to confirm the fidelity of mutagenesis.

**Figure 7:**
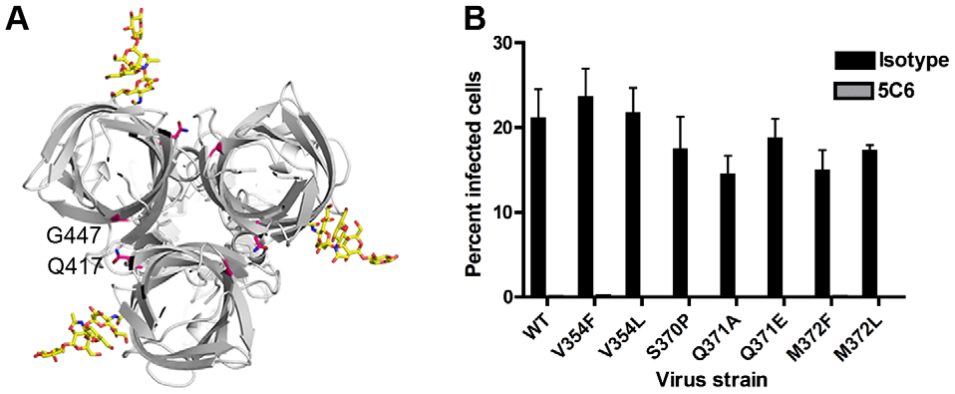
T1L σ1 point-mutant viruses are neutralized by mAb 5C6. (**A**) Top-view of T1L σ1 (gray) in complex with the GM2 glycan (yellow). Residues Q417 and G447, which are altered in mAb 5C6-resistant mutants and likely form part of the 5C6 epitope, are shown in stick representation in pink. (**B**) Wildtype and mutant viruses were incubated with conformation-specific T1L σ1-specific mAb 5C6 for 1 h, and the virus-antibody mixture was adsorbed to L cells for 1 h. Cells were washed twice with PBS, and fresh medium was added. After incubation at 37°C for 20 h, cells were fixed, and reovirus antigen was detected by indirect immunofluorescence. Nuclei were stained with DAPI. The percentage of infected cells in three fields of view per well was determined. The data shown are the mean infectivity per well from three independent experiments each performed in triplicate. Error bars represent S.E.M. *P*<0.001, as determined by two-tailed Student's *t* test for all virus strains.

We thought it possible that mutations within the putative carbohydrate-binding site might result in diminished infectivity in MEFs due to impaired glycan engagement or some other impairment in viral fitness. To eliminate the latter possibility and normalize infectious units for the virus strains tested, we used L cells, which do not require sialylated glycan engagement to support infection, likely due to an abundance of JAM-A on the cell surface. Unlike our findings with MEFs, neither neuraminidase treatment of cells (Figure 1) nor pretreatment of virus with GM2 (data not shown) altered T1L infectivity in L cells. To determine whether the mutant σ1 proteins are properly folded, we tested the conformation-sensitive monoclonal antibody (mAb) 5C6 for the capacity to inhibit mutant virus infection of L cells. Neutralization-resistant T1L mutants selected by mAb 5C6 have alterations at Q417 and G447 in T1L σ1 [43]. These residues are located at the upper part of the T1L σ1 head domain, close to the intersubunit interface (Figure 7A). An antibody that recognizes these residues likely binds a trimeric conformer of the T1L σ1 head and thus indicates the presence of properly folded and assembled σ1 trimers. Preincubation with mAb 5C6 significantly diminished the capacity of wildtype and mutant T1L viruses to infect L cells (Figure 7B), suggesting that the σ1 head domain of the mutants is recognized by mAb 5C6 and not grossly misfolded.

**Figure 8:**
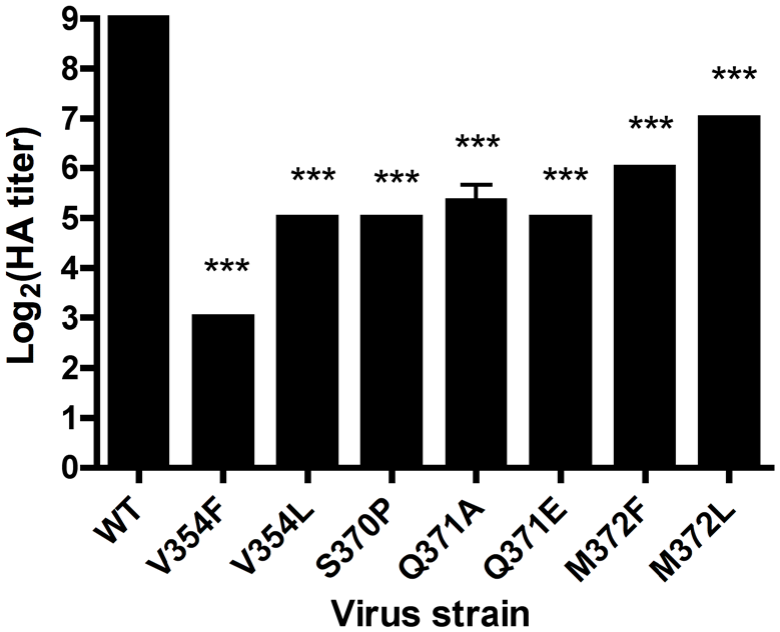
Hemagglutination by σ1 mutant viruses. Purified virions of the strains shown (10^11^ particles/well) were serially diluted 1∶2 in PBS in 96-well U-bottom plates. Human erythrocytes were washed several times with PBS, resuspended to a concentration of 1% (vol/vol) in PBS, added to virus-containing wells, and incubated at 4°C for 3 h. Results are expressed as log_2_ (HA titer). HA titer is defined as 10^11^ particles divided by the number of particles/HA unit. One HA unit is the particle number sufficient to produce hemagglutination. *** *P*<0.001, as determined by one-way Anova followed by Bonferroni's multiple comparison test.

**Figure 9:**
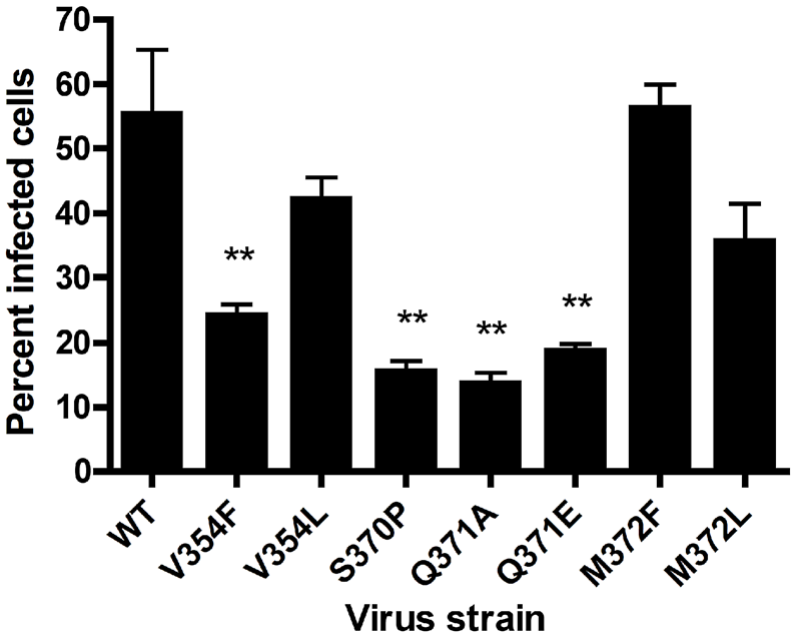
Infectivity of σ1 mutant viruses in MEFs. Monolayers of MEFs were adsorbed with the strains shown at an MOI of 1 FFU/field (as titered in L cells) at room temperature for 1 h. Cells were washed twice with PBS, and fresh medium was added. After incubation at 37°C for 20 h, cells were fixed, and reovirus antigen was detected by indirect immunofluorescence. Nuclei were stained with DAPI. The percentage of infected cells in three fields of view per well was determined. The results are from a representative experiment of three experiments performed with triplicate wells. Error bars represent standard deviations. **, *P*<0.01, as determined by two-tailed Student's *t* test.

To test whether the σ1 point mutants have impaired glycan binding, we quantified the capacity of wildtype and mutant viruses to agglutinate human erythrocytes (Figure 8), a property linked to carbohydrate binding [28]. All of the mutants had a significant defect in hemagglutination, with alterations of V354, S370, and Q371 showing the greatest impairment. To determine whether the point mutants have an altered capacity to infect cells in a carbohydrate-dependent fashion, we quantified infectivity in MEFs, which require carbohydrate binding for optimal infection (Figure 1). MEFs were inoculated with wildtype and mutant viruses at an MOI of 1 FFU/cell for each virus as equilibrated in assays using L cells. The V354F, S370P, Q371A, and Q371E mutants displayed a significant defect in infectivity in MEFs (Figure 9). Taken together, these data suggest that residues V354, S370, and Q371, which flank the carbohydrate-binding site of T1L σ1, are required for functional engagement of the GM2 glycan.

## Discussion

Although all known reovirus serotypes utilize JAM-A as a receptor, they display striking differences in viral tropism and spread. These differences segregate with the S1 gene, which encodes the σ1 attachment protein [7]. The σ1 residues that interact with JAM-A are conserved among the serotypes [25], and serotype-dependent tropism in the CNS is observed in JAM-A-null mice [11]. These observations suggest that serotype-dependent differences in host disease are attributable to σ1 engagement of cell-surface receptors other than JAM-A.

T3D σ1 binds to sialic acid using residues in its body domain, interacting with α2,3, α2,6, and α2,8-linked sialic acid in a similar manner [19], [27]. Although hemagglutination data [28] and lectin-based studies [30] demonstrate that T1L interacts with α2,3-linked sialic acid, neither the identity of the specific glycan nor the molecular basis of T1L-glycan interactions was known. In this study, we found that T1L uses the GM2 glycan as a functional receptor, which is the first identification of a specific glycan recognized by any reovirus serotype.

Hemagglutination assays have been used in many previous studies of reovirus-glycan interactions [27]–[29]. Reovirus displays serotype-dependent hemagglutination profiles. Type 1 reoviruses agglutinate human but not bovine erythrocytes, whereas type 3 reoviruses preferentially agglutinate bovine erythrocytes and agglutinate human erythrocytes less efficiently [29]. These observations suggest that the glycan-binding sites of type 1 and type 3 reovirus are distinct, a hypothesis that is now confirmed by this study and that of Reiter, et al [19]. Analysis of the respective crystal structures sheds light on the potential species differences in hemagglutination behavior. Whereas human erythrocytes express the Neu5Ac form of sialic acid [44], bovine cells express mostly Neu5Gc and less Neu5Ac [45]. The additional hydroxyl group of Neu5Gc would face a hydrophobic pocket in the type 1 σ1 glycan-binding site, making a favorable interaction unlikely. In contrast, the type 3 σ1 binding site likely could accommodate either Neu5Ac or Neu5Gc (D.M. Reiter and T. Stehle, unpublished data).

The GM2 glycan binds to the head domain of T1L σ1 and not, as predicted earlier, to the body region of the protein [27]. It is possible that cell-surface structures in addition to glycans contribute to hemagglutination by type 1 reovirus and this may explain why the chimeric σ1 proteins used in the earlier study had diminished, but not abolished, hemagglutination capacity. Alternatively, disruption of the neck domain of σ1 in the chimeric proteins used in the previous study [27] might have altered the conformation of the glycan-binding domain in the head.

Inspection of the carbohydrate-binding site reveals that the two terminal sugar moieties of the branched GM2 glycan, Neu5Ac and GalNAc, contact the protein, explaining the observed specificity of T1L σ1 for this receptor. Most of the contacts are contributed by Neu5Ac, which is wedged into a cleft between β-strands B and C at the side of the σ1 head, while the GalNAc docks onto a shallow protein surface using van der Waals interactions.

Although the GM3 oligosaccharide is also able to bind T1L σ1 in solution, infectivity studies indicate that GM2 is the preferred glycan receptor for T1L reovirus. While preincubation with either GM2 or GM3 oligosaccharides resulted in diminished infectivity of MEFs, the GM2 glycan blocked infectivity more efficiently and in a dose-dependent fashion. The “extra” GalNAc moiety of GM2 is likely responsible for the selectivity of T1L σ1 for this glycan. At only 41 Å^2^, the surface area in T1L σ1 buried by interactions with GalNAc is very small compared to the 284 Å^2^ surface buried by contacts with Neu5Ac in the same complex (Table S4), but the small additional interactions are nevertheless expected to mediate higher-affinity binding of the GM2 glycan compared with GM3, which lacks GalNAc. In addition, due to its branched structure, the GM2 glycan has less conformational freedom in solution than the linear GM3 molecule [41], which may also facilitate interactions with the virus. Entropy furthermore favors binding of the branched GM2 glycan over the linear GM3 molecule. In support of this idea, limited conformational freedom of the branched glycan GM1 is essential for its selective engagement by cholera toxin over related compounds [46]. Therefore, the branched sequence of the GM2 glycan sequence is preferred over the linear sequence of GM3.

Interactions between T1L σ1 and GM2 are primarily comprised of hydrogen bonds between the sugar molecule and backbone atoms of the protein. Nevertheless, we were able to identify residues required for functional glycan engagement by introducing mutations into the glycan-binding site. All mutants displayed impaired hemagglutination capacity, with mutations altering V354, S370, and Q371 having the greatest effect (Figure 8). Mutations affecting these same residues resulted in the greatest defect in infectivity of MEFs (Figure 9). Residue V354 flanks a hydrophobic pocket into which the methyl group of the *N*-acetyl chain of Neu5Ac inserts. Mutation of V354 to phenylalanine impairs infectivity of MEFs, while mutating the residue to leucine had a less dramatic effect. Changing S370 to proline introduces a protruding and rigid ring structure, which is expected to create steric hindrance within the glycan-binding pocket (Figure 5). Q371 likely forms a hydrogen bond with the carboxyl group of Neu5Ac. In the point mutants Q371E and Q371A, this hydrogen bond would be lost, which would lead to reduced ligand binding and, in the case of Q371E, electrostatic repulsion.

**Figure 10:**
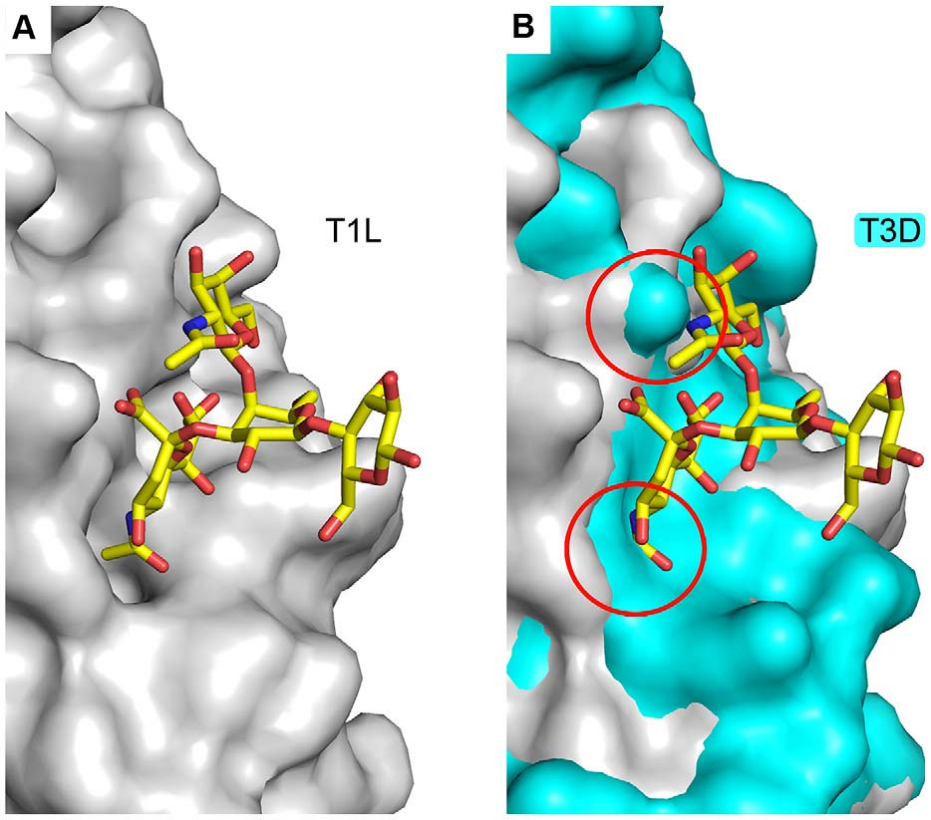
The head domain of T3D σ1 does not bind Neu5Ac. (**A**) Surface representation of T1L σ1 depicted in gray. (**B**) SSM superposition of T1L (gray) and T3D (cyan) σ1. The GM2 glycan is shown in stick representation (colors as in Figure 4) in both panels. Clashes between the carbohydrate and T3D σ1 are highlighted with red circles in panel B. Both the Neu5Ac and GalNAc moieties of the GM2 oligosaccharide would clash with T3D σ1 residues.

Interestingly, for the mutants S370P, and Q371A, the residue in T1L σ1 was changed to the corresponding residue in T3D σ1. Structural data suggest that the T1L glycan-binding pocket does not exist in T3D (Figure 10), which likely explains the serotype-dependent inhibition of infection by GM2 (Figure 3). Collectively, these data suggest that residues V354, S370, and Q371, which flank the carbohydrate-binding site of T1L σ1, are important for recognition and engagement of the GM2 glycan despite the predominant role of main-chain interactions in the crystallographic model.

**Figure 11:**
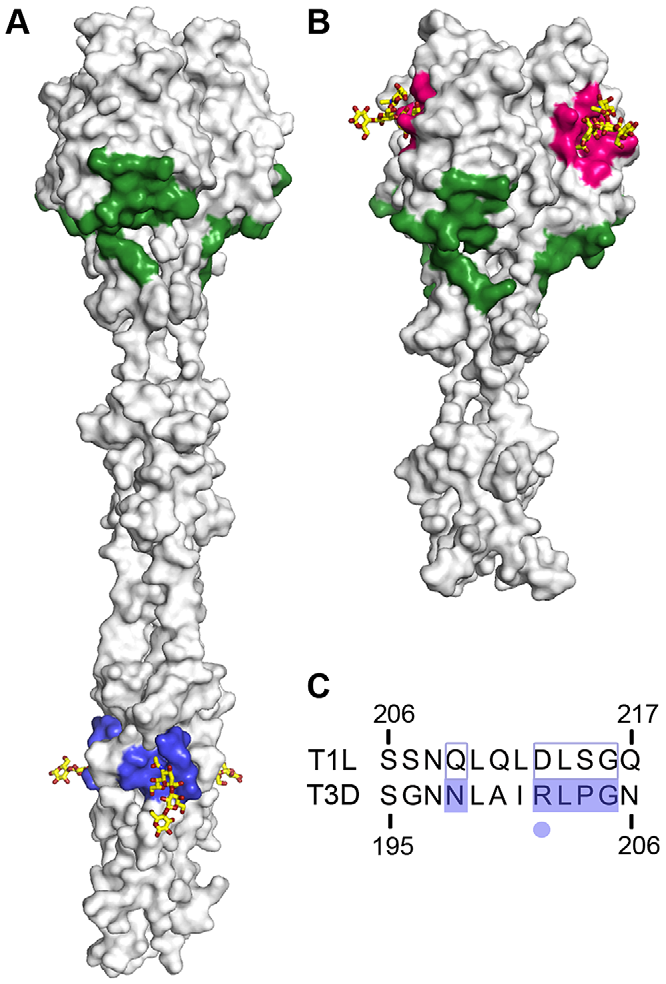
Comparison of the receptor-binding sites of T1L and T3D σ1. Surface representations of (**A**) T3D σ1 in complex with the GM3 glycan (PDB accession code 3S6X) and (**B**) T1L σ1 in complex with the GM2 glycan. The carbohydrates are shown in stick representation and colored as in Figure 4. The JAM-A-binding sites are highlighted in green, and the carbohydrate-binding sites in T1L and T3D σ1 are depicted in pink and blue, respectively. (**C**) Sequence alignment of the carbohydrate-binding site in T1L and T3D σ1. Residues required for carbohydrate engagement in T3D σ1 are highlighted in blue. Residue R202, which forms a central interaction with Neu5Ac in T3D σ1, is marked with a blue dot.

The GM2-binding site in T1L σ1 is distinct from the site of JAM-A binding, and we think that T1L σ1 can bind both receptors, perhaps in a sequential manner (Figure 11A, B). The N-terminal D1 domain of human JAM-A is not glycosylated [47]. Therefore, the glycan receptor must be an independent entity. Reovirus engagement of host cells is likely a multistep process in which interactions with glycans function in adhesion strengthening [33]. We anticipate that the virus first encounters cell-surface GM2 and binds with relatively low affinity (in line with the NMR data) and then binds JAM-A with high affinity [20], [48], followed by integrin-mediated uptake [49]. This model is supported by the finding that glycan binding is required for T1L infection of MEFs, which express modest levels of JAM-A, and dispensable in L cells, which display significantly higher levels of JAM-A expression. Glycan binding also can function independently of JAM-A engagement, as the relatively modest infectivity of JAM-A-null MEFs can be further reduced by neuraminidase treatment (data not shown). Furthermore, it is possible that the glycan functions with unknown receptors in the host or serves as the sole cell-surface molecule used by T1L in some tissues. The function of adhesion-strengthening and the interactions or lack thereof between GM2 and other reovirus receptors is an important topic for future research.

The precise tissue distribution of GM2 is not completely understood, but the glycan is a component of the mammalian nervous system [50]–[52]. In mice, T1L reovirus infects ependymal cells and causes hydrocephalus [8], [53]. The presence of GM2 in the brain provides an attractive explanation for the use of this coreceptor by T1L. Because ganglioside expression may differ in cell types that serve as targets for reovirus infection in vivo, there may be cells in which one glycan or another predominates as a T1L coreceptor. Type 3 reoviruses differing only in the capacity to engage cell-surface glycans display marked differences in tropism [54], [55]. We anticipate that glycan binding also functions in the pathogenesis of type 1 reovirus infections, which is an area of current investigation in our laboratories.

Reovirus is being tested in clinical trials as an oncolytic adjunct to conventional cancer therapy. Some tumor cells have altered ganglioside expression compared with untransformed cells, and some overexpress GM2 [56]–[58]. Humanized antibodies directed against GM2 prevent the formation of organ metastases in mice with small-cell lung cancer [59]. It is possible that ganglioside overexpression in tumor cells alters the susceptibility of certain cancers to reovirus infection. Understanding the molecular basis of reovirus-glycan interactions might improve the design of effective oncolytics.

Although T1L and T3D reoviruses bind sialylated glycans as receptors using their σ1 proteins, the locations of the respective carbohydrate-binding sites differ substantially (Figure 11A, B). The T1L σ1 glycan-binding site resides in the head domain. In contrast, the T3D σ1 glycan-binding site is in the N-terminal part of the body domain, close to the midpoint of the σ1 molecule. Structure and sequence comparisons show that the head of T3D σ1 would not be capable of engaging Neu5Ac-based receptors because the carbohydrate-binding site of the T1L σ1 head is blocked in T3D σ1 (Figures 5C, 10). It also is unlikely that the region of T1L σ1 corresponding to the T3D σ1 glycan-binding site would interact with sialic acid. T3D σ1 residue Arg202 forms critical interactions with Neu5Ac and, in T1L σ1, there is an aspartate instead of an arginine at the equivalent position. The negatively charged aspartate side chain would probably repel Neu5Ac and, thus, carbohydrate engagement at this site is impeded (Figure 11C). The different locations of the carbohydrate-binding sites contrast with the conserved interactions of both σ1 proteins with JAM-A. The JAM-A-binding sites of both T1L and T3D σ1 proteins are located at the base of the head domain, and interactions between σ1 and JAM-A are similar in both serotypes [25], [26]. Assuming that both protein- and carbohydrate-binding sites are accessible for both serotype 1 and serotype 3 reoviruses, it is possible that the mechanisms of attachment are not conserved between the reovirus serotypes, which may contribute to the observed differences in viral tropism and spread.

## Materials and Methods

### T1L σ1 protein expression and purification

Construct σ1_long_ comprises the three most C-terminal predicted β-spirals of T1L σ1 and the head domain (amino acids 261–470). Construct σ1_short_ comprises the most C-terminal predicted β-spiral of T1L σ1 and the head domain (amino acids 300–470). Expression and purification of T1L σ1_long_ and T1L σ1_short_ were facilitated by attaching a trimeric version of the GCN4 leucine zipper [60], [61] to the N-terminus of the σ1 sequence, similar to the strategy we used to express T3D σ1 [19]. The σ1 construct was cloned into the pQE-80L expression vector (Qiagen), which includes a non-cleavable N-terminal His_6_-tag. The protein was expressed in *E. coli* Rosetta 2 (DE3) (Novagen) by autoinduction at 20°C for 48 to 72 h. Bacteria were lysed using an EmulsiFlex (Avestin) homogenizer and purified via Ni-affinity chromatography (His-Trap FF column, GE Healthcare). The fusion protein was eluted from the column, and the protein solution was desalted using a PD10 desalting column (GE Healthcare). The GCN4 domain and the His_6_-tag were removed from the fusion protein using 1 µg trypsin per mg protein at 20°C for 4 h. The resultant products were subjected to size-exclusion chromatography (Superdex 200) to remove the tags, trypsin, and other minor impurities.

Undigested versions of both constructs were used for glycan array screening. STD NMR experiments were performed using σ1_long_. Both constructs were used for structural analysis. Uncleaved σ1_short_ yielded crystals diffracting to 2.6 Å resolution. This higher resolution structure was used as a reference model for refinement of the lower-resolution structures of cleaved σ1_long_ in complex with the GM2 or GM3 glycan.

### Glycan microarray analyses

Microarrays were composed of lipid-linked oligosaccharide probes, neoglycolipids (NGLs) and glycolipids, robotically printed on nitrocellulose-coated glass slides at 2 and 7 fmol per spot using a non-contact instrument, and analyses were performed as described [62], [63]. For analysis of T1L σ1_long_, the results of 124 oligosaccharide probes (5 non-sialylated and 119 sialylated, Glycosciences Array Set 40–41), at 5 fmol per spot are shown in Figure S1 and Table S1. For the analysis of T1L σ1_short_, a different version of the microarray (in house designation Ganglioside Dose Response Array set 1) was used; results of the 21 ganglioside-related probes (Table S2) each arrayed at four levels: 0.3, 0.8, 1.7 and 5.0 fmol/spot, are shown in Figure 2A.

For the initial analysis of His-tagged T1L σ1_long_, the protein was incubated with mouse monoclonal anti-poly-histidine (Ab1) and biotinylated anti-mouse IgG antibodies (Ab2) (both antibodies from Sigma) at a ratio of 4∶2∶1 (by weight). The σ1_long_-antibody complexes were prepared by preincubating Ab1 with Ab2 at ambient temperature for 15 min, followed by addition of His-tagged T1L σ1_long_ and incubation on ice for 15 min. The σ1_long_-antibody complexes were diluted in 5 mM HEPES (pH 7.4), 150 mM NaCl, 0.3% (v/v) Blocker Casein (Pierce), 0.3% (w/v) bovine serum albumin (Sigma), 5 mM CaCl_2_ and 40 mM imidazole (referred to as HBS-Casein/BSA-imidazole), to provide a final σ1_long_ concentration of 150 µg/ml, and overlaid onto the arrays at 20 °C for 2 h. Binding was detected using Alexa Fluor 647-labeled streptavidin (Molecular Probes) at 1 µg/ml. Microarray data analyses and presentation were facilitated using dedicated software [64].

For the analyses of His-tagged T1L σ1_short_, different assay conditions were evaluated with and without complexation (not shown). The condition selected as optimal was without precomplexation. His-tagged σ1_short_ was diluted in HBS-Casein/BSA-imidazole, overlaid at 300 µg/ml, followed by incubation with Ab1 and Ab2 (each at 10 mg/ml, precomplexed at ambient temperature for 15 min). Binding was detected using Alexa Fluor 647-labeled streptavidin.

### Crystallization, x-ray structure determination, and refinement

Crystals of uncleaved σ1_short_ formed in 0.1 M MES/imidazole (pH 6.5), 10% PEG 4000, 20% glycerol, 0.02 M sodium formate, 0.02 M ammonium acetate, 0.02 M trisodium citrate, 0.02 M sodium potassium L-tartrate, 0.02 M sodium oxamate at 4°C using the sitting-drop-vapor-diffusion method. No additional cryoprotection was necessary. Crystals of σ1_long_ formed in 0.1 M Na cacodylate (pH 6.0–6.6), 1.2–1.5 M (NH_4_)_2_SO_4_ at 4°C using the sitting-drop-vapor-diffusion method. For preparation of complexes, these crystals were transferred to 20 mM GM2 or GM3 oligosaccharide (Elicityl) in the crystallization solution for 5–10 min. Prior to flash-freezing, the crystals were transferred to a solution containing 0.1 M Na cacodylate, 1.34 M (NH_4_)_2_SO_4_, 25% glycerol, and 20 mM GM2 or GM3 glycan.

The crystals belonged to space group P3_2_21 and contained one trimer in the asymmetric unit. A complete data set was collected at the Swiss Light Source, beamline X06SA. XDS was used to index and scale the reflection data [65]. The structure was determined by molecular replacement with Phaser (CCP4) [66],[67] using the coordinates of T1L σ1 derived from the previously determined T1L σ1-JAM-A complex structure as a search model [26]. Manual model building was carried out using coot [68]. Structural refinement was performed using Refmac5 (CCP4) [69], Phenix [70], and autoBUSTER [71],[72].

Inspection of the 2F_o_-F_c_ maps for the structures of the T1L σ1-glycan complexes revealed clear, unambiguous electron density for most of the GM2 and GM3 oligosaccharides at a 1.5 σ contour level. The glycans also were visible in difference electron density maps. The unbiased electron density maps in Figures 4, 6, and S3 show the initial F_o_-F_c_ maps of the T1L σ1-GM2 and T1L σ1-GM3 glycan complexes obtained after molecular replacement using the previously solved structure of unliganded T1L σ1. The carbohydrates were included in the model at this point. Refinement of the ligands was performed using the CCP4 library and user-defined constraints. Structure images were created using PyMOL [73]. Coordinates and structure factors of both complexes have been deposited in the Protein Data Bank with accession codes 4GU3 (T1L-σ1-GM2 glycan complex) and 4GU4 (T1L σ1-GM3 glycan complex).

### Sequence and structural analysis

Sequence alignments were performed using T-Coffee [74] and analyzed using Jalview [75], [76]. Structure alignments were calculated by secondary-structure matching (SSM) superposition in coot [77]. The Ramachandran plot was generated with Rampage (CCP4) [78]. Buried surface areas were calculated using AreaImol (CCP4) [79], [80].

### STD NMR spectroscopy

NMR spectra were recorded using 3 mm tubes and a Bruker AVIII-600 spectrometer equipped with a room temperature probe head at 283 K and processed with TOPSPIN 3.0 (Bruker). Samples containing 1 mM GM2 or GM3 glycan (Elicityl), 20 mM potassium phosphate (pH 7.4), and 150 mM NaCl with and without 20 µM T1L σ1 were used for the STD NMR measurements and the frequency control, respectively. Samples were prepared in D_2_O, and no additional water suppression was used to preserve the anomeric proton signals. The sample without protein also was used for spectral assignment. The off- and on-resonance irradiation frequencies were set to −30 ppm and 7.3 ppm, respectively. The irradiation power of the selective pulses was 57 Hz, the saturation time was 2 s, and the total relaxation delay was 3 s. A 50 ms continuous-wave spin-lock pulse with a strength of 3.2 kHz was employed to suppress residual protein signals. A total number of 512 scans were recorded. A total of 10,000 points were collected, and spectra were multiplied with a Gaussian window function prior to Fourier transformation. Spectra were referenced using HDO as an internal standard [81].

### Cells

Spinner adapted murine L cells were grown in suspension culture in Joklik's minimum essential medium (Lonza) supplemented to contain 5% fetal bovine serum (FBS) (Gibco), 2 mM L-glutamine, 100 U/ml penicillin, 100 µg/ml streptomycin (Invitrogen), and 25 ng/ml amphotericin B (Sigma-Aldrich). MEFs were generated from C57/BL6 mice at embryonic day 13.5 as described [82]. MEFs were maintained in Dulbecco's modified Eagle's minimum essential medium (DMEM) (Gibco) supplemented to contain 10% FBS, 2 mM L-glutamine, 100 U/ml penicillin, 100 µg/ml streptomycin, 1X MEM nonessential amino acids (Sigma-Aldrich), 20 mM HEPES, and 0.1 mM 2-mercaptoethanol (Sigma-Aldrich). Cells at passages 3–6 were used in this study.

### Viruses and plasmid-based reovirus rescue

Viruses were generated using plasmid-based reverse genetics [42], [83]. BHK-T7 cells (5×10^5^) were seeded in 60 mm tissue-culture dishes (Corning) and allowed to incubate at 37°C overnight. OptiMEM (Invitrogen) (0.75 ml) was mixed with 53.25 µl TransIT-LT1 transfection reagent (Mirus) and incubated at RT for 20 min. Plasmid constructs representing cloned gene segments from the T1L genome, pT7S1 T1L, pT7S2 T1L, pT7L3S3 T1L, pT7S4 T1L, pT7M1 T1L, pT7L1M2 T1L, and pT7L2M3 T1L were mixed into the OptiMEM/TransIT-LT solution. Equal amounts of each plasmid were added for a total of 17.75 µg DNA. The plasmid-transfection solution was added to BHK-T7 cells and incubated for 3–5 days. Following two freeze-thaw cycles, recombinant viruses were isolated by plaque purification using L-cell monolayers [84]. Purified virions were generated using second-passage L cell-lysate stocks. Viral particles were Freon-extracted from infected cell lysates and layered onto 1.2 to 1.4 g/cm^3^ CsCl gradients and centrifuged at 62,000×g for 18 h. Bands were collected and dialyzed exhaustively in virion-storage buffer as described [12], [85]. To generate mutant viruses, resides V354, S370, Q371, and M372 in the S1 gene plasmid were altered by QuickChange (Stratagene) site-directed mutagenesis. S1 gene sequences were confirmed using the OneStep RTPCR kit (Qiagen), gene-specific primers, and viral dsRNA extracted from infected L cells (RNAeasy, Qiagen). Primer sequences for mutagenesis and sequencing are available from the corresponding authors by request. Sanger sequencing was performed using purified PCR products (Gene Hunter and Vanderbilt Sequencing Core). Genotypes were confirmed by electrophoresis of viral particles in 4-to-20% gradient sodium dodecyl sulfate polyacrylamide gels stained with ethidium bromide and visualized by UV illumination [86]. Particle concentrations were determined using the conversion 1 AU_260_ = 2.1×10^12^ particles [85]. Viral titers were quantified by plaque assay [84] or fluorescent focus assay [33].

### Antibodies

Reovirus polyclonal immunoglobulin G (IgG) raised against T1L and T3D was used to stain for reovirus antigen [87]. Alexa-488 conjugated goat anti-rabbit antibody (Invitrogen) was used as a secondary antibody. Monoclonal rat anti-mouse JAM-A (Abcam, clone H202-106) was used to stain for JAM-A expression followed by goat anti-rat secondary antibody conjugated to Alexa-488 (Invitrogen). Conformation-sensitive neutralizing mAb 5C6 specific for T1L [43], [88] was used in neutralization assays as described [89].

### Infectivity studies

L cells (10^5^) or MEFs (5×10^4^) were incubated in 24-well plates (Costar) at 37°C overnight. To evaluate the importance of sialic acid engagement in T1L infection, cell monolayers were treated with 100 mU/ml of *A. ureafaciens* neuraminidase diluted in PBS (MP Biomedicals, LLC) or PBS alone (mock) at RT for 1 h prior to virus adsorption at an MOI of 1 PFU/cell in L cells or 100 PFU/cell (as titered in L cells) in MEFs. Following incubation at RT for 1 h, the inoculum was removed, and cells were washed twice with PBS and incubated at 37°C for 20 h. Cells were fixed in methanol and visualized by indirect immunofluorescence [33] with the addition of a DAPI stain to quantify cell nuclei. Cells were blocked in PBS supplemented to contain 5% bovine serum albumin (BSA) (Sigma). Infected cells were detected by staining with reovirus polyclonal antiserum diluted 1∶1000 and secondary Alexa-488 goat anti-rabbit Ig 1∶1000 (Invitrogen). Nuclei were quantified using DAPI (1∶1000). All antibodies were diluted in PBS supplemented to contain 0.5% Triton X-100. Infectivity studies were performed in triplicate wells. Three fields of view per well were quantified using the Axiovert 200 fluorescence microscope (Carl Zeiss).

To determine the effect of soluble glycans on viral infectivity, virus was incubated with various concentrations of GM2 or GM3 glycan (Elicityl) at room temperature for 1 h. The virus-glycan mixture was adsorbed to MEFs (MOI of 100 PFU/cell as titered on L cells) at room temperature for 1 h. The cells were washed twice, and infectivity was determined by immunofluorescence assay.

### Flow cytometry

To determine the relative amount of JAM-A on L cells and MEFs, 5×10^5^ cells were stained with rat anti-mouse JAM-A at a dilution of 1∶200 followed by staining with Alexa-488 labeled goat anti-rat Ig at 1∶1000. All staining was done in PBS supplemented to contain 2% FBS. Fluorescence was measured using an LSRII (BD, Vanderbilt University Flow Cytometry Shared Resource). Mean fluorescence intensity of a forward and side scatter gated population was determined using FlowJo software.

### Hemagglutination assay

Purified reovirus virions (10^11^ particles) were distributed into 96-well U-bottom microtiter plates (Costar) and serially diluted twofold in 0.05 ml of PBS. Human type O erythrocytes (Vanderbilt Blood Bank) were washed twice with PBS and resuspended at a concentration of 1% (vol/vol). Erythrocytes (0.05 ml) were added to wells containing virus particles and incubated at 4°C for 3 h. A partial or complete shield of erythrocytes on the well bottom was interpreted as a positive HA result; a smooth, round button of erythrocytes was interpreted as a negative result. HA titer is expressed as 10^11^ particles divided by the number of particles/HA unit. One HA unit equals the number of particles sufficient to produce HA.

### Statistical analysis

Statistical analysis was performed using Prism (Graphpad). Two-tailed Student's *t* tests were used for all infectivity studies. The hemagglutination assays were analyzed using a one-way Anova followed by a Bonferroni's correction. *P* values of less than 0.05 were considered to be statistically significant.

## Supporting Information

Figure S1 **Glycan microarray analyses of T1L-σ1_long_ using a microarray of 124 lipid-linked oligosaccharide probes.** Numerical scores of the binding signals are means of duplicate spots at 5 fmol/spot (with error bars). The various types of terminal sialic acid linkage are indicated by the colored panels as defined at the bottom of the figure. Error bars are all relatively large due to the low fluorescent signals. The list of probes and their sequences and binding scores are provided in Table S1. The X indicates an artifact on the slide giving a false signal resulting in a large error bar.
(TIF)

Figure S2 **STD NMR spectroscopy of T1L σ1 with GM2 and GM3 oligosaccharide.** (**A**) Chemical structure of the GM2 glycan. Protons that receive saturation upon binding to T1L σ1 are color-coded according to the corresponding STD NMR spectrum in Figure 2C. (**B**) T1L σ1 binds to the GM3 glycan in solution. STD NMR experiment of T1L σ1 and the GM3 oligosaccharide. Upper spectrum: ^1^H spectrum of the GM3 glycan alone; middle: STD spectrum of T1L σ1 and the GM3 glycan; and lower spectrum: STD spectrum of the GM3 oligosaccharide alone to ensure that no direct excitation of the glycan takes place. A schematic drawing of GM3 is provided in the upper left corner.
(TIF)

Figure S3 **CaRp analysis of the T1L σ1-GM2 complex.** CaRp analysis (Carbohydrate Ramachandran plot, www.glycosciences.de) of the three GM2 oligosaccharide molecules in the T1L σ1-GM2 complex. A schematic of the GM2 oligosaccharide is included with the three glycosidic bonds numbered. The structure of one GM2 glycan molecule and its unbiased F_o_-F_c_ map at 3.0 σ contour level for 2.0 Å are shown at the bottom right.
(TIF)

Figure S4 **T1L σ1 binds Neu5Ac of the GM2 glycan and the GM3 glycan at the same site.** SSM superposition of the T1L σ1-GM2 complex (yellow) and the T1L σ1-GM3 complex (cyan). The protein chains are shown as ribbon tracings, and the Neu5Ac moieties of the GM2 and GM3 glycan are depicted in stick representation in yellow and cyan, respectively. They superimpose with an r.m.s.d. value of 0.76 Å.
(TIF)

Table S1 **Oligosaccharide probes used in the initial glycan microarray analyses, sorted by sialyl linkage and backbone sequence, and the binding signals (means of the fluorescence intensity at ∼5 fmol/probe spot) of T1L-σ1_long_.**
(DOC)

Table S2 **List of probes and sequences included in the ganglioside dose-response array set.**
(DOC)

Table S3 **Dihedral angles of the glycosidic linkages of the three GM2 oligosaccharides bound to T1L σ1.**
(DOC)

Table S4 **T1L σ1 surface areas buried by GM2 and GM3 in the σ1-glycan complex structures.**
(DOC)
