# Supplementary figures and images for: The GM2 Glycan Serves as a Functional Coreceptor for Serotype 1 Reovirus
Source: PLoS Pathog. 2012 Dec 6;8(12):e1003078. doi: 10.1371/journal.ppat.1003078 (PMC3516570; doi:10.1371/journal.ppat.1003078)

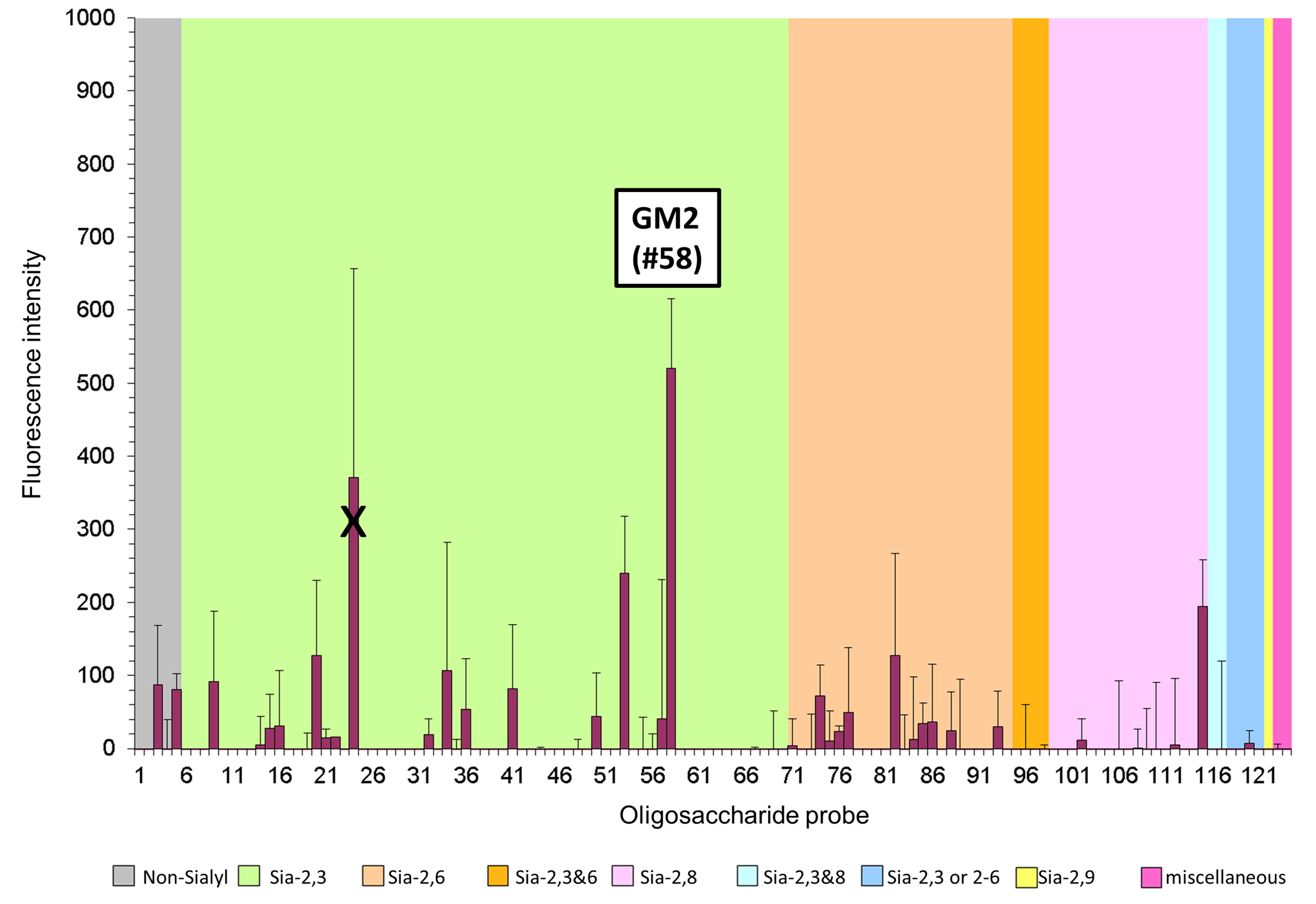

Supplement: Figure S1 — Glycan microarray analyses of T1L-σ1long using a microarray of 124 lipid-linked oligosaccharide probes. Numerical scores of the binding signals are means of duplicate spots at 5 fmol/spot (with error bars). The various types of terminal sialic acid linkage are indicated by the colored panels as defined at the bottom of the figure. Error bars are all relatively large due to the low fluorescent signals. The list of probes and their sequences and binding scores are provided in Table S1. The X indicates an artifact on the slide giving a false signal resulting in a large error bar. (TIF) [file ppat.1003078.s001.tif]

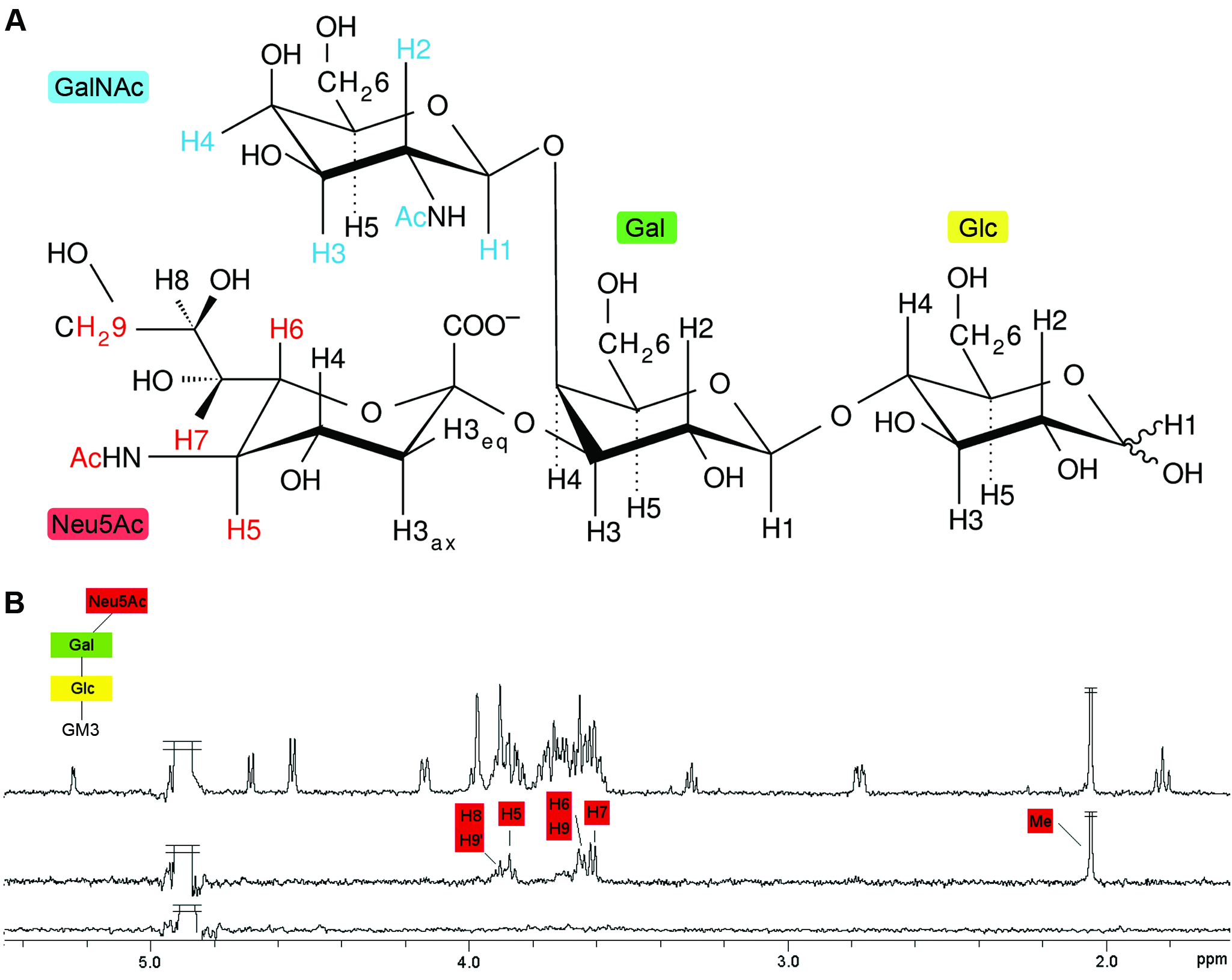

Supplement: Figure S2 — STD NMR spectroscopy of T1L σ1 with GM2 and GM3 oligosaccharide. (A) Chemical structure of the GM2 glycan. Protons that receive saturation upon binding to T1L σ1 are color-coded according to the corresponding STD NMR spectrum in Figure 2C. (B) T1L σ1 binds to the GM3 glycan in solution. STD NMR experiment of T1L σ1 and the GM3 oligosaccharide. Upper spectrum: 1H spectrum of the GM3 glycan alone; middle: STD spectrum of T1L σ1 and the GM3 glycan; and lower spectrum: STD spectrum of the GM3 oligosaccharide alone to ensure that no direct excitation of the glycan takes place. A schematic drawing of GM3 is provided in the upper left corner. (TIF) [file ppat.1003078.s002.tif]

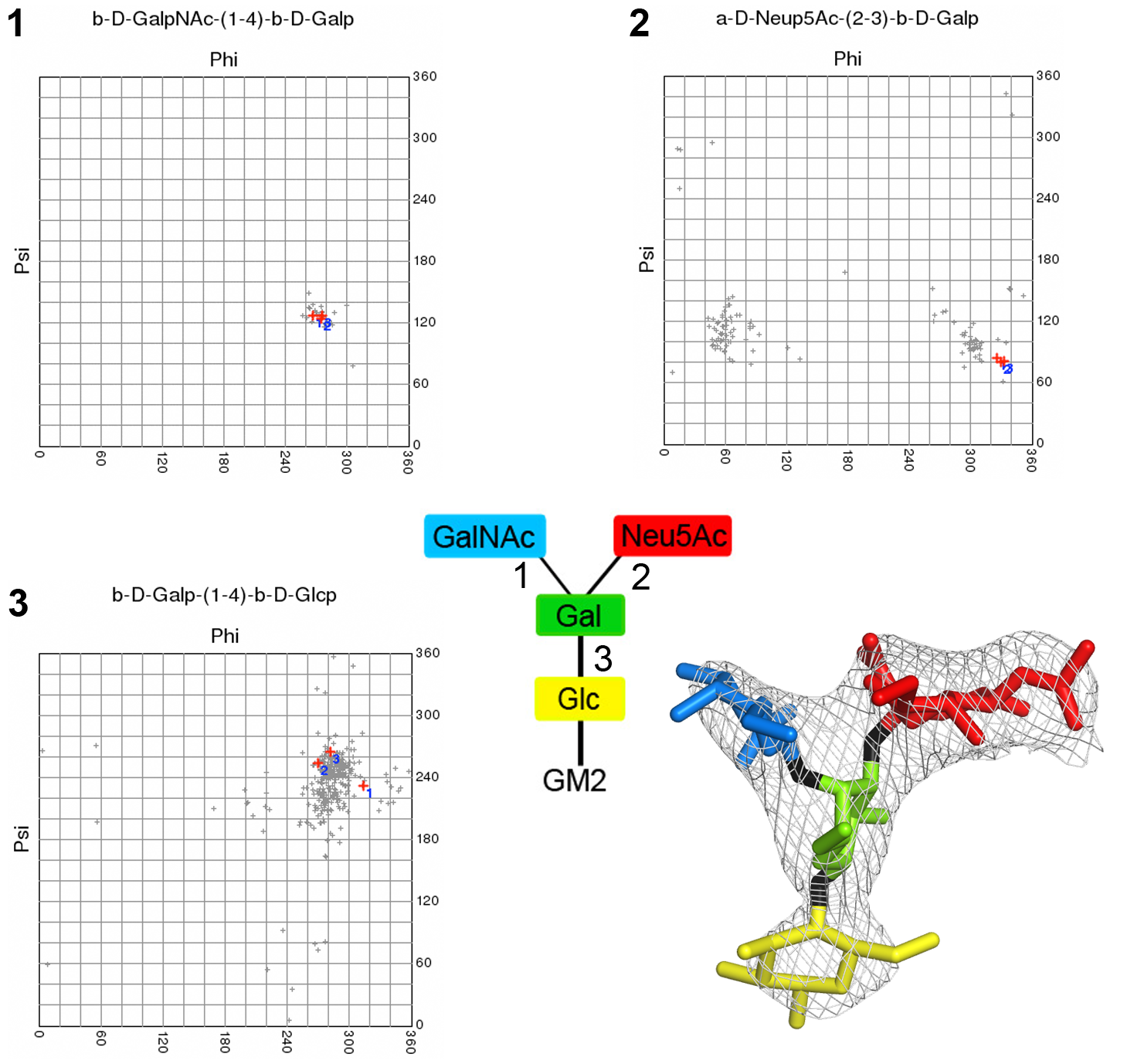

Supplement: Figure S3 — CaRp analysis of the T1L σ1-GM2 complex. CaRp analysis (Carbohydrate Ramachandran plot, www.glycosciences.de) of the three GM2 oligosaccharide molecules in the T1L σ1-GM2 complex. A schematic of the GM2 oligosaccharide is included with the three glycosidic bonds numbered. The structure of one GM2 glycan molecule and its unbiased Fo-Fc map at 3.0 σ contour level for 2.0 Å are shown at the bottom right. (TIF) [file ppat.1003078.s003.tif]

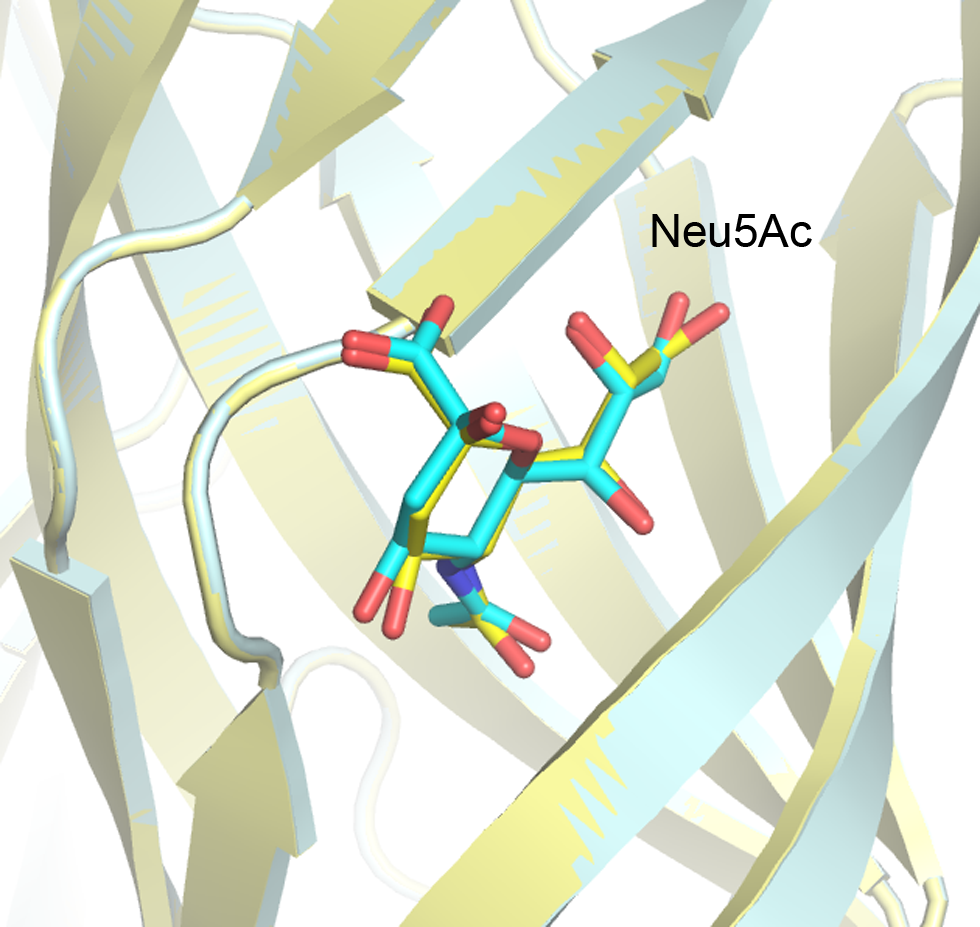

Supplement: Figure S4 — T1L σ1 binds Neu5Ac of the GM2 glycan and the GM3 glycan at the same site. SSM superposition of the T1L σ1-GM2 complex (yellow) and the T1L σ1-GM3 complex (cyan). The protein chains are shown as ribbon tracings, and the Neu5Ac moieties of the GM2 and GM3 glycan are depicted in stick representation in yellow and cyan, respectively. They superimpose with an r.m.s.d. value of 0.76 Å. (TIF) [file ppat.1003078.s004.tif]
