# Supplementary material for: The GM2 Glycan Serves as a Functional Coreceptor for Serotype 1 Reovirus
Source: PLoS Pathog. 2012 Dec 6;8(12):e1003078. doi: 10.1371/journal.ppat.1003078 (PMC3516570; doi:10.1371/journal.ppat.1003078)
Supplement: Table S1 — Oligosaccharide probes used in the initial glycan microarray analyses, sorted by sialyl linkage and backbone sequence, and the binding signals (means of the fluorescence intensity at ∼5 fmol/probe spot) of T1L-σ1long. (DOC) [file ppat.1003078.s005.doc]

**Table S1.
Oligosaccharide probes used in the initial glycan microarray analyses, sorted by sialyl linkage and backbone sequence, and the binding signals (means of the fluorescence intensity at ~5 fmol/probe spot) of T1L-σ1_long_.**

| **Position** | **Probe^a^** | **Structure^b^** | **Fluorescence**  **intensity** | **Error** |
| --- | --- | --- | --- | --- |
| 1 | LNT | Galß-3GlcNAcß-3Galß-4Glc | -**^c^** | - |
| 2 | LNnT | Galß-4GlcNAcß-3Galß-4Glc | - | - |
| 3 | LNFP-III | Galß-4GlcNAcß-3Galß-4Glc  │  Fucα-3 | 88 | 81 |
| 4 | LNH | Galß-4GlcNAcß-6   │   Galß-4Glc   │  Galß-3GlcNAcß-3 | - | 40 |
| 5 | NA2 | Galß-4GlcNAcß-2Manα-6   │   Manß-4GlcNAcß-4GlcNAc  │  Galß-4GlcNAcß-2Manα-3 | 81 | 22 |
| 6 | NeuAcα-(3')Lac | NeuAcα-3Galß-4Glc | - | - |
| 7 | NeuAcα-(3')Lac-AO | NeuAcα-3Galß-4Glc-AO | - | - |
| 8 | Neu4,5Ac-(3')Lac | Neu4,5Acα-3Galß-4Glc | - | - |
| 9 | Neu4,5Ac-(3')Lac-AO | Neu4,5Acα-3Galß-4Glc-AO | 92 | 97 |
| 10 | GSC-17 | NeuAcα-3Galß-4Glcß-Cer36 | - | - |
| 11 | GSC-197 | KDNα-3Galβ-4Glcß-Cer28 | - | - |
| 12 | GSC-198 | KDNα-3Galβ-4Glcß-Cer34 | - | - |
| 13 | GSC-75 | (4-deoxy)NeuAcα-3Galß-4Glcß-Cer36 | - | - |
| 14 | GSC-76 | (7-deoxy)NeuAcα-3Galß-4Glcß-Cer36 | 6 | 39 |
| 15 | GSC-77 | (8-deoxy)NeuAcα-3Galß-4Glcß-Cer36 | 28 | 46 |
| 16 | GSC-153 | (4,8-deoxy)NeuAcα-3Galß-4Glcß-Cer36 | 31 | 77 |
| 17 | GSC-51 | (9-deoxy)NeuAcα-3Galß-4Glcß-Cer36 | - | - |
| 18 | GSC-78 | (4-OMe)NeuAcα-3Galß-4Glcß-Cer36 | - | - |
| 19 | GSC-79 | (9-OMe)NeuAcα-3Galß-4Glcß-Cer36 | - | 21 |
| 20 | Neuα-(3')Lac | Neuα-3Galß-4Glc | 128 | 103 |
| 21 | Neuα-(3')Lac-AO | Neuα-3Galß-4Glc-AO | 15 | 13 |
| 22 | NeuAcß-(3')Lac | NeuAcß-3Galß-4Glc | 16 | - |
| 23 | NeuAcß-(3')Lac-AO | NeuAcß-3Galß-4Glc-AO | - | - |
| 24 | NeuAcα-(3')LN | NeuAcα-3Galß-4GlcNAc | 371 | 286 |
| 25 | NeuAcα-(3')LN-AO | NeuAcα-3Galß-4GlcNAc-AO | - | - |
| 26 | SA(3')-Lea-Tri | NeuAcα-3Galß-3GlcNAc  │  Fucα-4 | - | - |
| 27 | GSC-513 | Neu5,9Acα-3Galß-3GlcNAcß-C30  │  Fucα-4 | - | - |
| 28 | GSC-511 | Neu5,9Acα-3Galß-4GlcNAcß-C30  │  Fucα-3 | - | - |
| 29 | GSC-105 | NeuAcα-3Galß-4GlcNAcß-3Galß-Cer36  │  Fucα-3 | - | - |
| 30 | GSC-341 | KDNα-3Galβ-4GlcNAcβ-3Galß-C30  │   Fucα-3 | - | - |
| 31 | GSC-177 | NeuGcα-3Galβ-4GlcNAcβ-3Galß-Cer36   │   Fucα-3 | - | - |
| 32 | LSTa | NeuAcα-3Galß-3GlcNAcß-3Galß-4Glc | 19 | 23 |
| 33 | GSC-147 | KDNα-3Galβ-3GlcNAcβ-3Galβ-4Glcß-Cer36 | - | - |
| 34 | GSC-396 | NeuGcα-3Galβ-3GlcNAcβ-3Galβ-4Glcß-C30 | 107 | 176 |
| 35 | Sialylparagloboside | NeuAcα-3Galß-4GlcNAcß-3Galß-4Glcß-Cer | - | 12 |
| 36 | GSC-31 | NeuAcα-3Galß-4GlcNAcß-3Galß-4Glcß-Cer36 | 54 | 69 |
| 37 | GSC-516B | Neuα-3Galß-4GlcNAcß-3Galß-4Glcß-Cer36  │  SU-6 | - | - |
| 38 | SA(3')-LNFP-II | NeuAcα-3Galß-3GlcNAcß-3Galß-4Glc   │   Fucα-4 | - | - |
| 39 | GSC-64 | NeuAcα-3Galß-4GlcNAcß-3Galß-4Glcß-Cer36   │   Fucα-3 | - | - |
| 40 | SA(3')-LNFP-III | NeuAcα-3Galß-4GlcNAcß-3Galß-4Glc  │  Fucα-3 | - | - |
| 41 | GSC-472 | Neuα-3Galß-4GlcNAcß-3Galß-4Glcß-Cer36  │  Fucα-3 | 82 | 87 |
| 42 | GSC-149 | KDNα-3Galβ-4GlcNAcβ-3Galβ-4Glcß-Cer36  │   Fucα-3 | - | - |
| 43 | GSC-268 | SU-6  │ NeuAcα-3Galß-4GlcNAcß-3Galß-4Glcß-Cer36  │   Fucα-3 | - | - |
| 44 | GSC-268 deNAc | SU-6  │ Neuα-3Galß-4GlcNß-3Galß-4Glcß-Cer36  │   Fucα-3 | - | 2 |
| 45 | GSC-269 | SU-6  │ NeuAcα-3Galß-4GlcNAcß-3Galß-4Glcß-Cer36  │   Fucα-3 | - | - |
| 46 | GSC-406 | SU-6  │ Neuα-3Galß-4GlcNAcß-3Galß-4Glcß-Cer36  │   Fucα-3 | - | - |
| 47 | GSC-270 | SU-6 SU-6   │ │ NeuAcα-3Galß-4GlcNAcß-3Galß-4Glcß-Cer36  │  Fucα-3 | - | - |
| 48 | MSMFLNH | Galß-4GlcNAcß-6  │ │  Fucα-3 Galß-4Glc  │ NeuAcα-3Galß-3GlcNAcß-3 | - | 13 |
| 49 | GSC-221 | NeuAcα-3Galß-4GlcNAcß-3Galß-4GlcNAcß-3Galß-4Glcß-Cer36  │  Fucα-3 | - | - |
| 50 | GSC-220 | NeuAcα-3Galß-4GlcNAcß-3Galß-4GlcNAcß-3Galß-4Glcß-Cer36  │ │  Fucα-3 Fucα-3 | 44 | 60 |
| 51 | C4U | NeuAcα-3Galβ-4GlcNAcβ-3Galβ-3GlcNAc  │ │ │  SU-6 SU-6 SU-6 | - | - |
| 52 | FucC4U | Fucα-3  │ NeuAcα-3Galβ-4GlcNAcβ-3Galβ-3GlcNAc  │ │ │  SU-6 SU-6 SU-6 | - | - |
| 53 | A2F(2-3) | NeuAcα-3Galß-4GlcNAcß-2Manα-6 Fucα-6  │ │  Manß-4GlcNAcß-4GlcNAc  │ NeuAcα-3Galß-4GlcNAcß-2Manα-3 | 240 | 78 |
| 54 | GM4 | NeuAcα-3Galβ-Cer | - | - |
| 55 | Haematoside | NeuAcα-3Galß-4Glcß-Cer | - | 43 |
| 56 | GM3 | NeuAcα-3Galß-4Glcß-Cer | - | 20 |
| 57 | GM3(Gc) | NeuGcα-3Galß-4Glc-Cer | 41 | 190 |
| 58 | GM2 | GalNAcβ-4Galβ-4Glcβ-Cer   │   NeuAcα-3 | 520 | 96 |
| 59 | GSC-193 | GalNAcβ-4Galβ-4Glcß-Cer36   │   KDNα-3 | - | - |
| 60 | GM1b | NeuAcα-3Galβ-3GalNAcß-4Galß-4Glcß-Cer* | - | - |
| 61 | GM1 | Galβ-3GalNAcβ-4Galβ-4Glcβ-Cer   │   NeuAcα-3 | - | - |
| 62 | GM1-penta | Galβ-3GalNAcβ-4Galβ-4Glc   │   NeuAcα-3 | - | - |
| 63 | GM1(Gc) | Galβ-3GalNAcβ-4Galβ-4Glcβ-Cer   │   NeuGcα-3 | - | - |
| 64 | GM1(Gc)-penta | Galβ-3GalNAcβ-4Galβ-4Glc   │   NeuGcα-3 | - | - |
| 65 | GD1a | NeuAcα-3Galß-3GalNAcß-4Galß-4Glcß-Cer  │  NeuAcα-3 | - | - |
| 66 | GD1a-hexa | NeuAcα-3Galß-3GalNAcß-4Galß-4Glc  │  NeuAcα-3 | - | - |
| 67 | GalNAc-GD1a(Ac,Gc) | GalNAcß-4Galß-3GalNAcß-4Galß-4Glcß-Cer   │ │  NeuGcα-3 NeuAcα-3  GalNAcß-4Galß-3GalNAcß-4Galß-4Glcß-Cer   │ │  NeuAcα-3 NeuGcα-3 | - | 2 |
| 68 | GSC-195 | KDNα-3Galβ-3GalNAcβ-4Galβ-4Glcß-Cer36   │   KDNα-3 | - | - |
| 69 | GSC-154 | NeuAcα-3Galß-4GlcNAcß-6Galß-4Glcß-Cer36  │  Fucα-3 | - | 51 |
| 70 | GSC-441 | NeuAcα-3Galß-4GlcNAcß-6GalNAcα-3Galß-4Glcß-C30 | - | - |
| 71 | GSC-144 | KDNα-6Galß-Cer36 | 4 | 38 |
| 72 | NeuAcα-(6')Lac | NeuAcα-6Galß-4Glc | - | - |
| 73 | NeuAcα-(6')Lac-AO | NeuAcα-6Galß-4Glc-AO | - | 47 |
| 74 | GSC-61 | NeuAcα-6Galß-4Glcß-Cer36 | 73 | 42 |
| 75 | Neuα-(6')Lac | Neuα-6Galß-4Glc | 11 | 42 |
| 76 | Neuα-(6')Lac-AO | Neuα-6Galß-4Glc-AO | 23 | 8 |
| 77 | NeuAcß-(6')Lac | NeuAcß-6Galß-4Glc | 50 | 89 |
| 78 | NeuAcß-(6')Lac-AO | NeuAcß-6Galß-4Glc-AO | - | - |
| 79 | NeuAcα-(6')LN | NeuAcα-6Galß-4GlcNAc | - | - |
| 80 | Neu5,9Ac-(6')LN | Neu5,9Acα-6Galß-4GlcNAc | - | - |
| 81 | LSTb | Galβ-3GlcNAcβ-3Galβ-4Glc  │ NeuAcα-6 | - | - |
| 82 | LSTc | NeuAcα-6Galβ4-GlcNAcβ3-Galβ4-Glc | 127 | 140 |
| 83 | SA(6')-LNFP-VI | NeuAcα-6Galß-4GlcNAcß-3Galß-4Glc  │  Fucα-3 | - | 46 |
| 84 | GSC-97 | NeuAcα-6Galß-4GlcNAcß-3Galß-4Glcß-Cer36  │  Fucα-3 | 12 | 86 |
| 85 | MSLNH | NeuAcα-6Galß-4GlcNAcß-6  │  Galß-4Glc  │  Galß-3GlcNAcß-3 | 34 | 28 |
| 86 | MSLNnH-I | Galß-4GlcNAcß-6  │  Galß-4Glc  │ NeuAcα-6Galß-3GlcNAcß-3 | 36 | 79 |
| 87 | DSLNnH | NeuAcα-6Galß-4GlcNAcß-6  │   Galß-4Glc  │  NeuAcα-6Galß-4GlcNAcß-3 | - | - |
| 88 | MFMSLNnH | Galß-4GlcNAcß-6  │ │  Fucα-3 Galß-4Glc  │ NeuAcα-6Galß-3GlcNAcß-3 | 24 | 54 |
| 89 | A2(2-6) | NeuAcα-6Galß-4GlcNAcß-2Manα-6  │   Manß-4GlcNAcß-4GlcNAc  │ NeuAcα-6Galß-4GlcNAcß-2Manα-3 | - | 95 |
| 90 | GSC-442 | GalNAcβ-4Galβ-4Glcß-Cer36   │   NeuAcα-6 | - | - |
| 91 | GSC-68 | NeuAcα-6Galß-3GalNAcß-4Galß-4Glcß-Cer36 | - | - |
| 92 | GSC-155 | Galβ-3GalNAcβ-4Galβ-4Glcß-Cer36   │  NeuAcα-6 | - | - |
| 93 | GSC-107 | NeuAcα-6Galβ-3GalNAcβ-4Galβ-4Glcß-Cer36   │   NeuAcα-6 | 30 | 49 |
| 94 | GSC-70 | NeuAcα-6Galß-6GalNAcß-4Galß-4Glcß-Cer36 | - | - |
| 95 | DSLNT | NeuAcα-3Galß-3GlcNAcß-3Galß-4Glc   │  NeuAcα-6 | - | - |
| 96 | A3 | NeuAcα-3Galß-4GlcNAcß-2Manα-6  │   Manß-4GlcNAcß-4GlcNAc  │ NeuAcα-3Galß-4GlcNAcß-4Manα-3  │  NeuAcα-6Galß-4GlcNAcß-2 | - | 60 |
| 97 | GSC-118 | NeuAcα-3Galβ-3GalNAcβ-4Galβ-4Glcß-Cer36   │   NeuAcα-6 | - | - |
| 98 | DST | NeuAcα-3Galß-3GalNAc  │  NeuAcα-6 | - | 5 |
| 99 | GSC-437 | NeuAcα-8NeuAcα-8NeuAcα-3Galβ-4Glcß-Cer36 | - | - |
| 100 | GD3 | NeuAcα-8NeuAcα-3Galß-4Glcß-Cer | - | - |
| 101 | GD3-tetra | NeuAcα-8NeuAcα-3Galß-4Glc | - | - |
| 102 | GD3-tetra-AO | NeuAcα-8NeuAcα-3Galß-4Glc-AO | 11 | 30 |
| 103 | GD2 | GalNAcß-4Galß-4Glcß-Cer  │ NeuAcα-8NeuAcα-3 | - | - |
| 104 | GD1b | Galß-3GalNAcß-4Galß-4Glcß-Cer  │ NeuAcα-8NeuAcα-3 | - | - |
| 105 | GQ1b | NeuAcα-8NeuAcα-3Galβ-3GalNAcβ-4Galβ-4Glcβ-Cer  │  NeuAcα-8NeuAcα-3 | - | - |
| 106 | SA2(α8) | NeuAcα-8NeuAc | - | 93 |
| 107 | SA3(α8) | NeuAcα-8NeuAcα-8NeuAc | - | - |
| 108 | SA4(α8) | NeuAcα-8NeuAcα-8NeuAcα-8NeuAc | 1 | 27 |
| 109 | SA5(α8) | NeuAcα-8NeuAcα-8NeuAcα-8NeuAcα-8NeuAc* | - | 55 |
| 110 | SA6(α8) | NeuAcα-8NeuAcα-8NeuAcα-8NeuAcα-8NeuAcα-8NeuAc* | - | 91 |
| 111 | SA7(α8) | NeuAcα-8NeuAcα-8NeuAcα-8NeuAcα-8NeuAcα-8NeuAcα-8NeuAc* | - | - |
| 112 | SA8(α8) | NeuAcα-8NeuAcα-8NeuAcα-8NeuAcα-8NeuAcα-8NeuAc-8NeuAcα-8NeuAc* | 5 | 91 |
| 113 | SA9(α8) | NeuAcα-8NeuAcα-8NeuAcα-8NeuAcα-8NeuAcα-8NeuAc-8NeuAcα-8NeuAc-8NeuAcα* | - | - |
| 114 | SA10(α8) | NeuAcα-8NeuAcα-8NeuAcα-8NeuAcα-8NeuAcα-8NeuAc-8NeuAcα-8NeuAcα-8NeuAcα-8NeuAc* | - | - |
| 115 | SA11(α8) | NeuAcα-8NeuAcα-8NeuAcα-8NeuAcα-8NeuAcα-8NeuAc-8NeuAcα-8NeuAαc-8NeuAcα-8NeuAcα-8NeuAc* | 194 | 64 |
| 116 | GT1a | NeuAcα-8NeuAcα-3Galβ-3GalNAcβ-4Galβ-4Glcβ-Cer  │  NeuAcα-3 | - | - |
| 117 | GT1b | NeuAcα-3Galβ-3GalNAcβ-4Galβ-4Glcβ-Cer  │  NeuAcα-8NeuAcα-3 | - | 120 |
| 118 | SA(3/6)LNFP-I | NeuAcα-3/6Galß-3GlcNAcß-3Galß-4Glc  │  Fucα-2 | - | - |
| 119 | AGP-Bi-Ac2 | NeuAcα-Galß-4GlcNAcß-2Manα-6  │  Manß-4GlcNAcß-4GlcNAc  │ NeuAcα-Galß-4GlcNAcß-2Manα-3 | - | - |
| 120 | AGP-Bi-Gc2 | NeuGcα-Galß-4GlcNAcß-2Manα-6  │  Manß-4GlcNAcß-4GlcNAc  │ NeuGcα-Galß-4GlcNAcß-2Manα-3 | 7 | 17 |
| 121 | AGP-Bi-AcGc | NeuGcα-Galß-4GlcNAcß-2Manα-6  ? │  Manß-4GlcNAcß-4GlcNAc  │ NeuAcα-Galß-4GlcNAcß-2Manα-3 | - | - |
| 122 | GSC-96 | NeuAcα-9NeuAcα-3Galβ-4Glcß-Cer36 | - | - |
| 123 | NeuAc-AO | NeuAc-AO | - | 6 |
| 124 | NeuGc-AO | NeuGc-AO | - | - |

^a^The oligosaccharide probes are all lipid-linked, neoglycolipids (NGLs) or glycosylceramides.

^b^Unless otherwise specified, the NGLs are prepared from reducing oligosaccharides by reductive amination with the amino lipid, 1,2-dihexadecyl-*sn*-glycero-3-phosphoethanolamine (DHPE); AO, NGLs prepared from reducing oligosaccharides by oxime ligation with an aminooxy (AO) functionalized DHPE; Cer, natural glycolipids with various ceramide moieties; Cer36 and Cer42, synthetic glycolipids with ceramide having a total of 32 and 42 carbon atoms, respectively; C30, a synthetic lipid [2-(tetradecyl)hexadecanol] with 30 carbon atoms.

^c^ -, less than 1.

*Major component.
