# Supplementary material for: The GM2 Glycan Serves as a Functional Coreceptor for Serotype 1 Reovirus
Source: PLoS Pathog. 2012 Dec 6;8(12):e1003078. doi: 10.1371/journal.ppat.1003078 (PMC3516570; doi:10.1371/journal.ppat.1003078)
Supplement: Table S3 — Dihedral angles of the glycosidic linkages of the three GM2 oligosaccharides bound to T1L σ1. (DOC) [file ppat.1003078.s007.doc]

**Table S3.**

Dihedral angles of the glycosidic linkages of the three GM2 oligosaccharides bound to T1L σ1.

| **Glycosidic linkage** | **Chain** | **Dihedral angles (°)** | |
| --- | --- | --- | --- |
|  |  | **phi** | **psi** |
| β-GalNAc-(1-4)-β-Gal | A | 276.3 | 126.0 |
|  | B | 275.9 | 126.3 |
|  | C | 278.9 | 125.9 |
| α-Neu5Ac-(2-3)-β-Gal | A | 336.1 | 78.9 |
|  | B | 327.5 | 84.6 |
|  | C | 329.0 | 87.6 |
| β-Gal-(1-4)-β-Glc | A | 296.8 | 245.8 |
|  | B | 293.7 | 243.2 |
|  | C | 296.1 | 244.4 |
