# Supplementary material for: The GM2 Glycan Serves as a Functional Coreceptor for Serotype 1 Reovirus
Source: PLoS Pathog. 2012 Dec 6;8(12):e1003078. doi: 10.1371/journal.ppat.1003078 (PMC3516570; doi:10.1371/journal.ppat.1003078)
Supplement: Table S4 — T1L σ1 surface areas buried by GM2 and GM3 in the σ1-glycan complex structures. (DOC) [file ppat.1003078.s008.doc]

**Table S4.**

T1L σ1 surface areas buried by GM2 and GM3 in the σ1-glycan complex structures.

|  | **Buried surface area (Å^2^)** | |
| --- | --- | --- |
| **Molecule** | **T1L σ1-GM2** | **T1L σ1-GM3** |
| T1L σ1 | 247.5 | 197.9 |
| Carbohydrate | 330.8 | 299.1 |
| Neu5Ac | 284.0 | 299.1 |
| Gal | 5.8 | 0 |
| GalNAc | 40.8 | / |
